# Designing better input support programs: Lessons from zinc subsidies in Andhra Pradesh, India

**DOI:** 10.1371/journal.pone.0242161

**Published:** 2020-12-03

**Authors:** Shweta Gupta, Avinash Kishore, Muzna Fatima Alvi, Vartika Singh

**Affiliations:** 1 Environment and Production Technology Division, International Food Policy Research Institute, New Delhi, India; 2 South Asia Region Office, International Food Policy Research Institute, New Delhi, India; Ohio State University South Centers, UNITED STATES

## Abstract

India has one of the largest agricultural input support programs in the world, delivered in the form of subsidies to farmers, raising concerns about its sustainability. This paper evaluates the performance of one such support, the micronutrient subsidy program in the state of Andhra Pradesh (AP) and presents a case for providing this support in the form of direct cash transfers. Under the program, key soil micronutrients- zinc, boron, and gypsum were distributed free of cost to farmers living in micronutrient-deficient areas, with identification and targeting managed entirely by the state. We survey 1621 farmers, 61 agriculture extension officers, and 78 agriculture input dealers to assess the efficacy of the program and to identify bottlenecks preventing effective targeting, with a focus on zinc. We find that use of non-subsidized zinc is high in AP, and awareness of benefits of zinc and physical access to input dealer shops are significant predictors of zinc use. We argue that the free provision of micronutrients may have created demand among farmers, but there is little justification to continue subsidizing such a program at such high rates or resorting to public distribution. We find that micronutrient procurement and distribution has become a burden on extension staff and crowds out the private sector. Our analysis shows that the subsidy can benefit more farmers if it is channeled through the network of private fertilizer dealers. We use administrative data on budgetary outlays and digital soil maps to suggest fiscal redistribution in the form of direct cash transfers that may ensure more effective targeting at a lower cost to the state.

## Introduction

Soil nutrient degradation has been a significant problem for India, following years of imbalanced, nitrogen-heavy chemical fertilizer use. Distortionary direct subsidies have kept the relative price of urea low and have made other macro- and micronutrients much more expensive [1, 2]. Farmers, for that matter, have lacked awareness about soil quality and crop requirements that would prevent soil depletion [3–5]. Andhra Pradesh is one of the states with the highest use of nitrogen-based fertilizers, well above the national mean [6].

In 2015, when Soil Health Card (SHC) data for the state of Andhra Pradesh (AP) was released, it was found that the soil in large parts of the state was deficient in micronutrients. Around 40% to 49% of soil samples were deficient in zinc, 33% in boron, and 20% to 24% in sulfur [7]. To reduce these deficiencies, in 2015 the AP government initiated a program to provide a 50% subsidy for micronutrients. SHC data were used to identify areas critically deficient in micronutrients and farmers in the “deficient” areas could buy zinc, gypsum, and boron from state agricultural department outlets at reduced rates. In 2017–18, the subsidy was raised from 50% to 100%, making micronutrients freely available to eligible farmers [8]. Even after three years of free distribution of micronutrients, the state still has nutrient-deficient soil. Digital soil maps created using SHC data from 2016–17 show that 32% of cultivated land in the state is still zinc deficient. In the latest SHC data from 2019, the zinc-deficient area had increased to 40% of the cultivated land in 2017–18 [9]. A soil sample is considered zinc deficient if it has less than 0.6 parts per million (ppm) of zinc in it.

Although subsidies can provide an initial push to adopt new agricultural technologies and practices [10–13], a growing body of literature recognizes the unsustainable burden that they impose on the state, especially in India [14, 15]. Several factors play a role in the success of these support programs—liquidity constraints of the target population [16], crowding-out of commercial spending [17], and the importance of investments in agricultural extension and rural infrastructure [18, 19]. Recently, the question of replacing price subsidies with cash transfers has received greater attention [20].

The effectiveness of AP’s micronutrient subsidy scheme can be gauged using several measures, such as whether the scheme led to a reduced level of soil deficiency, whether there was increased awareness and uptake of micronutrient application by farmers, and whether there were yield gains from increased use of subsidized micronutrients. In this paper, we focus on zinc which accounts for nearly 70% of the total outlay of the micronutrient program and is a critical nutrient for paddy- the largest crop of the state.

What factors determine farmers’ uptake of micronutrients, particularly of zinc? What role can the private sector play in improving balanced nutrient application? What are the alternative ways of allocating resources more efficiently for better targeting? We explore these questions and assess the performance of the scheme using a mix of primary and secondary data. We use survey data from multiple stakeholders, digital soil maps, administrative data, and qualitative interviews for our analysis. Findings from our paper can thus help in formulating policies that can effectively target zinc deficiency in the state and in other parts of the country where zinc deficiency is common. This study can inform policymakers in India and beyond, as they transition from a system of generous but administratively cumbersome price subsidies to a system of better-targeted direct benefit transfers (DBT) and cash transfers.

## Importance of zinc for cereal crops

The current study is pursued by the International Food Policy Research Institute (IFPRI) under the Soil Intelligence Systems (SIS) program, in partnership with Bill and Melinda Gates Foundation (BMGF) [21]. The program aims to predict soil properties as well as to measure the crop response to nutrients, accounting for the role of macro-nutrients, weather, soil salinity and other related factors for cereal crops. As part of the program, we restrict our analysis to paddy crop which is a major cereal crop accounting for more than 50% (2.2 million ha) of the area under food grains and nearly 76% of total food grains produced in the state [22].

In addition to its complementarity with the use of major farm inputs like fertilizers, manure and irrigation, micronutrient balance in the soil is found to be largely dependent on the removal/depletion of micronutrients from soils by different crops. Among crops, cereals remove higher amount of nutrients compared to other crops like pulses and oilseeds, because the yield of cereals is 1.5 to 3 times higher than that of pulses or oilseeds [23]. Apart from that, cereal grains are also rich in antinutritive compounds, like phytates, which reduce the bioavailability of a key nutrient—Zn by forming insoluble Zn–phytate complexes [24]. Therefore, cereal grains represent a very poor Zn source in the diet. This becomes a cause of concern in a country where cereal based foods account for nearly 60% of energy requirement in urban and rural regions unlike other zinc-rich foods like animal sources [25].

We focus primarily on zinc, among various micronutrients covered in the scheme- zinc, boron, gypsum, since it assumes the greatest significance in exploiting the high yield potentials of modern crop varieties. Long-term and multilocation field experiments with rice and wheat have shown a high requirement for Zn in addition to NPK applications for maintaining high crop yields [26]. An application of zinc can reduce the phytic acid contents in paddy so that the crop retains its natural zinc content [27]. In India, zinc deficiency is considered the fifth most important yield-limiting nutrient (following N, P, K, and S) and in India’s lowland crops like rice, it is second only to N [28]. Being one of the major paddy-producing states in the country, AP therefore directly suffers from this problem. Even the micronutrient subsidy program allots nearly 70% of the total outlay to zinc alone since deficiency of zinc in AP is much higher than that of other micronutrients like boron manganese and copper [29]. As a result, agriculture practices that help in maintaining adequate nutrient levels in the crop and policies aimed to achieve that goal are essential.

## Institutional framework and data

The micronutrient subsidy scheme is managed by the AP Department of Agriculture. The Andhra Pradesh State Cooperative Marketing Federation (APMARKFED) serves as the nodal agency that invites tenders from various manufacturers and transporters for the supply and transportation of micronutrients. The APMARKFED buys products from the lowest bidders, and then helps transport the products of selected manufacturers to various transit destinations. Based on SHC program data, areas that are critically deficient in micronutrients are identified. Following random checks to verify the quality of the micronutrients, the zinc, gypsum, and boron are distributed to centralized locations in the districts and made available at various Primary Agricultural Cooperative Societies (PACS) at the village level, and in some cases, at various *Mandal* (block) Agricultural Offices. Presently, the state supplies three types of zinc (Zinc Sulfate 21%, Zinc Sulfate 33% (monohydrate), and Zinc Sulfate 12% (Zinc EDTA)), two variants of boron (Boron 10.5% and Boron 20%), and gypsum in bagged and bulk form [29]. This is the first-ever scheme to be launched by any state that is solely targeted on micronutrient uptake. The *Bhoochetna* scheme of Government of Karnataka government is the only comparison in which micronutrient subsidy was a major component [30]. What makes the current scheme different is that micronutrients are supplied at a 100% subsidy.

AP farmers practice two methods of zinc application: application of zinc directly in the soil, at or before planting (also called basal application); and foliar application, where zinc is sprayed on the leaves during the vegetative phase of the crop by mixing zinc with water. For basal application, a dosage of 50 kilograms (kg) per hectare (ha) of zinc sulfate 21% is recommended. For foliar application, the recommended application is 0.5 kg of Zinc EDTA per ha [7]. Of the two, the basal application is the most recommended and the most practiced method for applying zinc since it leads to better nutrient absorption in the soil.

As seen in Table 1, in the five years between 2014–15 and 2018–19, the Government of AP has spent Rs. 2.5 billion to distribute 38,433 tons of zinc for 3.9 million ha of land. The procurement and distribution process are managed by Multi-Purpose Extension Officers (MPEOs) who are *Mandal*-level (subdistrict) extension agents. The micronutrient scheme is only one of the many agricultural schemes that these government agents manage. The recruitment of MPEOs (as contractual workers) began in 2014–15, replacing the old position of *Adarsha Rythu* (or “model farmers”) in the state. These workers hold primary responsibility for the delivery of last-mile extension services to farmers. MPEOs ensure timely availability of the requisite quantities of micronutrients in villages, maintain records of stocks, provide feedback from farmers on the quality and effectiveness of these inputs, and conduct extension and information-spreading about the efficacy of micronutrients to encourage their use among farmers. An android application called “D-Krishi” is used to manage micronutrient sales. The app is Aadhar-enabled and contains all a farmer’s key details, including acreage and SHC information, which can be verified using farmers’ biometric information.

**Table 1:** Annual budget for micronutrient subsidy.

Year	Zinc sulfate distributed (metric tons)	Total area covered (in hectares)	Total subsidy value (in INR)
2014–15	3,600	287,545	122 million
2015–16	6,833	751,390	298 million
2016–17	6,613	818,077	484 million
2017–18	13,465	1,273,205	865 million
2018–19	7,922	730,426	703 million

*Source*: [8]

**Fig 1:**
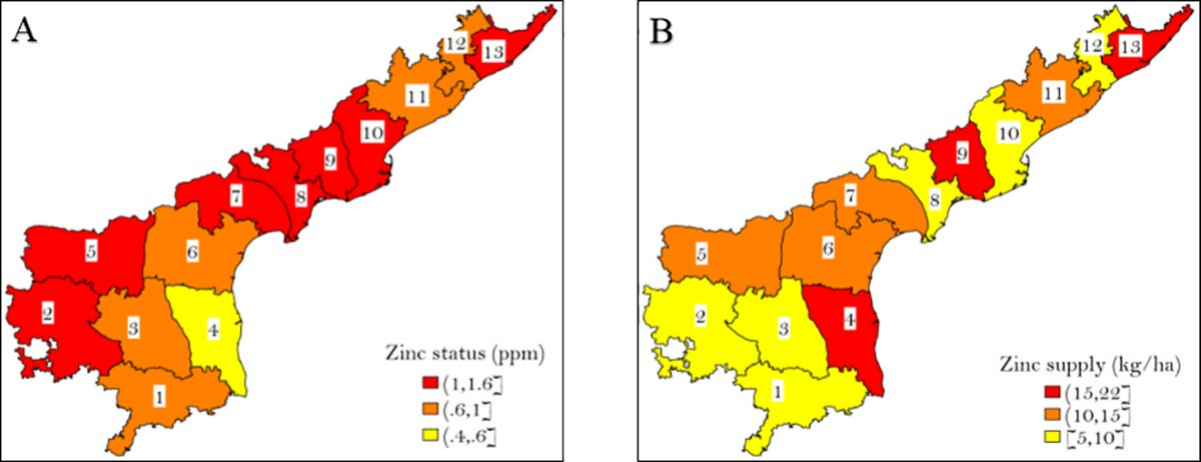
Soil zinc status (ppm) and the quantity of free zinc distributed in Andhra Pradesh, 2017–18. Source: Authors calculations. Shapefiles were accessed from open-access source GADM (https://gadm.org/index.html). The districts are numbered as follows: 1 = Chittoor, 2 = Anantapur, 3 = Kadapa, 4 = Nellore, 5 = Kurnool, 6 = Prakasam, 7 = Guntur, 8 = Krishna, 9 = West Godavari, 10 = East Godavari, 11 = Vishakhapatnam, 12 = Vizianagaram, 13 = Srikakulam. Panel A shows the district-wise zinc status in parts per million (ppm). These numbers are calculated using Soil Health Data from the second cycle (2017–18). Panel B shows the district-wise supply of zinc in 2017–18, in kg per hectare of the area covered. Area covered is a sum of area under paddy, maize, cotton, and other crops. These numbers are calculated using data from the zinc trials conducted by CIMMYT in 2017–18.

Although free micronutrients were to be provided only to farmers in deficit regions, there seems to be no correlation between the level of soil deficiency and the quantity of the micronutrients supplied across different districts of the state. For example, West Godavari and Srikakulam, two districts with relatively high levels of zinc in the soil, received more zinc per ha of land compared to Chittoor, Kadapa, and Vizianagaram, all areas where zinc deficiency is higher (Fig 1).

Apart from free public distribution by the government, there is a parallel commercial market for zinc, where private companies sell nonsubsidized formulations of zinc products to farmers through a network of single and multi-brand private retail shops. The commercial prices of zinc formulations are not regulated. In the private market, Zinc Sulfate 21% costs around INR 700–900 for 10 kg while a more expensive Zinc EDTA costs around INR 1,000 per kg, with significant variations across different brands. Fertilizer companies also independently undertake extension activities like organizing farmer field days, setting up demonstration plots, and traditional face-to-face interactions with farmers through private extension agents.

## Sampling and methodology

Our survey covered six (out of 13) districts in AP—Chittoor, East Godavari, West Godavari, Krishna, Nellore, and Srikakulam—that together cover nearly 63% of the total area under paddy in the state [22]. Using the probability proportional to size sampling method, 135 villages were selected out of 7000 villages in the state using E-panta data obtained from the Department of Agriculture, Government of AP. Within each village, 12 farming households were surveyed using simple random sampling from household lists obtained from village/block offices, thereby making a final sample of 1621 households. Along with farmers, surveys of 78 agricultural input dealers -19 cooperative agriculture input dealers and 59 private fertilizer dealers- and 61 government extension workers, both MPEOs and Agricultural Extension Officers (AEOs) of the state were also conducted. The surveys were conducted in Rabi season, 2018–19 and collected data on cultivation practices, the use of chemical inputs including micronutrients over Kharif 2018 and Rabi 2018–19, and awareness about micronutrient benefits from farmers. The surveys of MPEO/AEOs and fertilizer dealers included questions on main roles and responsibilities and issues faced in the implementation of the micronutrient subsidy scheme.

Before conducting the final surveys, focus group discussions were conducted by the research team in 2 districts of Andhra Pradesh- East Godavari and Vishakhapatnam, in 2018 with relevant stakeholders, to understand the on-ground functioning of the micronutrient subsidy scheme. We also rely substantially on lessons learned from the experimental farm tests conducted by Centre for Maize and Wheat Improvement (CIMMYT) and consultations with experts from the state agricultural department and state agricultural university (ANGRAU). NEERMAN Research and Consultancy served as our data collection partner. The surveys were conducted digitally using SurveyCTO software. All three questionnaires were pretested before conducting actual surveys and translated into the local language- Telugu.

Besides the primary data, we also use data from the official SHC database to measure soil health and zinc deficiency in the state. These data are supplemented with data from digital soil maps created by CIMMYT. State budgetary documents and government notifications are used to study the scheme targeting method, calculate fiscal allocations, and make budget predictions. Data analysis was conducted using Stata version 16.

Ethics Statement- An Institutional Review Board approval for this study was obtained on 1^st^ December 2018 from IFPRI’s IRB. All members of the research team have undergone Research ethics and compliance training under the Collaborative Institutional Training Initiative (CITI). Additionally, verbal consent was obtained from all survey respondents before conducting the final surveys with them.

## Results

Of the farmers in our sample, 82% grew paddy as their primary crop while sugarcane, maize, pulses, oilseeds and vegetables were among others grown by 3%, 3%, 10%, 9% and 1% of the sampled farmers respectively. Nearly one-third of the sample (30%) were tenant farmers. All sampled farmers cultivated some crop in Kharif (monsoon) 2018 and 41% of them grew a second crop in Rabi (winter) 2018–19. Table 2 provides the descriptive statistics of farmers surveyed.

**Table 2:** Descriptive statistics of sample households.

Characteristics	Mean (Sd)/ Proportion
Age (years)	50.23 (12.35)
Male (%)	94.2
Has some formal education (%)	60.7
Land owned (acres)	2.30 (2.53)
Cultivated in Kharif 2018 (%)	96.55
Cultivated in Rabi 2018–19 (%)	41.21
Land cultivated in Kharif 2018 (acres)	3.26 (4.71)
Land cultivated in Rabi 2018–19 (acres)	3.64 (6.36)
Tenant farmer (%)	30.04
Primary crop Paddy in either season (%)	81.55
Applied zinc in either season (%)	44.91
Applied boron in either season (%)	3.89
Applied gypsum in either season (%)	13.33
Can identify zinc deficiency in soil (%)	46.88
Owns a Soil Health Card (%)	11.66
Caste:	
General Caste (%)	36.64
Scheduled Caste (%)	11.23
Scheduled Tribe (%)	7.4
Other Backward Class (%)	44.73
*Number of observations*	*1*,*621*

Note: Standard deviation in parentheses

### Role of information, awareness, and extension

Only 45% of farmers reported using zinc for any crop in the last 12 months. The use of boron and gypsum was found to be even lower- 4% and 13% respectively. Among zinc users, more than half (56%) had purchased it from commercial markets. Tenants are often left out of agricultural subsidies that are linked to land ownership records [31–33]. However, 12% of tenant farmers in our sample had also received and applied free zinc to their crops.

**Fig 2:**
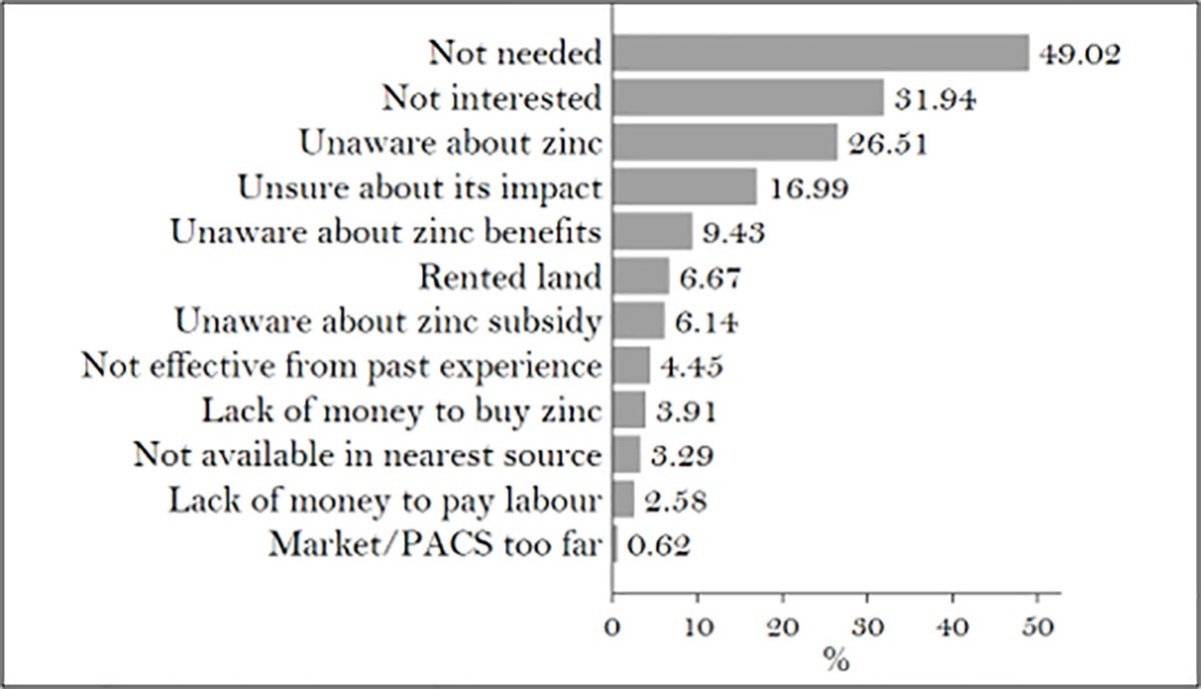
Reasons for non-usage of zinc. Source: Authors’ calculations.

Among the farmers who had not applied zinc, roughly half reported that they did not need zinc on their plots and stated that this was why they had not applied it. Many farmers in the sample also cited limited information and awareness about the benefits of zinc as reasons for non-application. A large proportion was unaware of zinc and its benefits (Fig 2), even though nearly 47% of the farmers reported that they could identify zinc deficiency in their soil. Issues of access to markets, labor, or credit might have been a reason for non-adoption, but these points did not emerge as major constraints in our survey. SHCs are supposed to be one of the major sources by which the farmers can gauge their soil’s nutrient deficiencies. However, in our sample, only 12% owned an SHC, and a fifth of those who had one did not understand its contents. 70% of the sampled farmers were unaware of the SHC scheme itself.

### Determinants of zinc use

To better understand what determines both the decision to use zinc and the amount of zinc, we use multivariate regression on our sample of farmers, controlling for a rich set of variables. We use a linear probability model to identify factors associated with the use of zinc, controlling for awareness about zinc as well as other farmer and village-level characteristics. Awareness about zinc is measured by farmers’ ability to identify zinc deficiency on their plots (see S1 Table for a description of variables included in the regression). Table 3 shows the results of this regression. Results in the first column are for the entire sample, whereas the second column is for paddy farmers.

**Table 3:** Determinants of zinc usage.

Variables	Used zinc in 2018–19	Used zinc in 2018–19
		(only paddy farmers)
Zinc Awareness = 1	0.340***	0.366***
	(-0.0277)	(-0.0309)
Land owned (acres)	0.0310***	0.0298**
	(-0.0116)	(-0.013)
Land owned squared (acres)	−0.00176*	−0.0019*
	(-0.001)	(-0.001)
Tenant = 1	0.0878***	0.0781**
	(-0.0321)	(-0.0331)
Educated = 1	0.0628**	0.0484
	(-0.0256)	(-0.0294)
Used Boron = 1	0.315***	0.334***
	(-0.0533)	(-0.0615)
Used Gypsum = 1	0.0121***	0.123**
	(-0.0402)	(-0.0476)
Distance from nearest shop (km)	−0.0115***	−0.0128***
	(-0.0039)	(-0.00426)
Cultivated Paddy in 2018–19 = 1	0.117***	–
	(-0.0358)	
District Fixed Effects	Yes	Yes
R- squared	0.235	0.232
Number of observations	1620	1321

Note: Standard errors in parentheses

***, **, and * denote significance at 1%, 5%, and 10%, respectively. We also controlled for farming experience (years), ownership of SHC data, interaction with MPEOs, and caste fixed effects. Standard errors are clustered around villages.

In our sample, an awareness of the benefits of zinc application has the strongest correlation with zinc use. Farmers who knew how to identify zinc deficiency were 34% more likely to use zinc in their plots. Larger landholders and literate farmers were more likely to use zinc. Surprisingly, tenant farmers (who often are left out of many agricultural subsidy programs such as crop insurance, *Kisan* credit cards for term agricultural loans, and direct cash transfers) are more likely to have used zinc than owner farmers. However, this positive correlation might also be attributable to the greater purchase of zinc rather than the use of free zinc. Distance from fertilizer shops also has a strong negative correlation with zinc usage, as one might expect, indicating that access or transportation costs could be a strong impediment to zinc use. Farmers living far from fertilizer shops also are likely to be those who live in relatively remote areas, which would imply lower access to information from both public extension workers and private companies and dealers. Many have found that the benefits of fertilizer subsidies do not reach small farmers and are mostly concentrated on large farmers [34, 35]. To check if this is true in our case, we conduct a similar regression as above but for free zinc users and purchased zinc users separately. Instead of using land as a continuous variable, land categories are included, namely marginal, small, semi-medium and medium farmers. We find that the probability of using free zinc is positively linked to farmland class but has no impact on the probability of using market zinc (see S2 Table for full results from this regression).

There is a strong argument in development debates that charging a positive price for a commodity is necessary to ensure its effective use [36]. For consumers, the price becomes the index of quality for new or unfamiliar products [37]—as is the case with zinc for many farmers in AP. Products that are free or low-priced may signal low quality, at least to new users, and may lead to low uptake if there is even a small amount of transaction cost involved in obtaining the “free” input. This could be one of the reasons why more than 50% of all farmers in our sample did not use zinc in their plots even when it is free and the probability of a farmer using it has a small but significant negative association with the distance to the fertilizer outlet. That said, the demand for a subsidized product is liable to drop drastically when its price is raised even slightly above zero [38–40]. In the case of zinc, however, this seems to be less of a concern. One in four farmers in our sample had purchased zinc from the market at full price and a simple contingent valuation exercise demonstrated that even the farmers who received free zinc were willing to pay INR 20–30 per kg for zinc. Although the exercise of eliciting the true willingness to pay for zinc could be misleading due to a tendency to anchor valuations around the subsidized price (here zero), in an incentive compatible experimental auction, Fishman and others found similar values of the willingness to pay for zinc among paddy growers in the state of Bihar [4].

**Fig 3:**
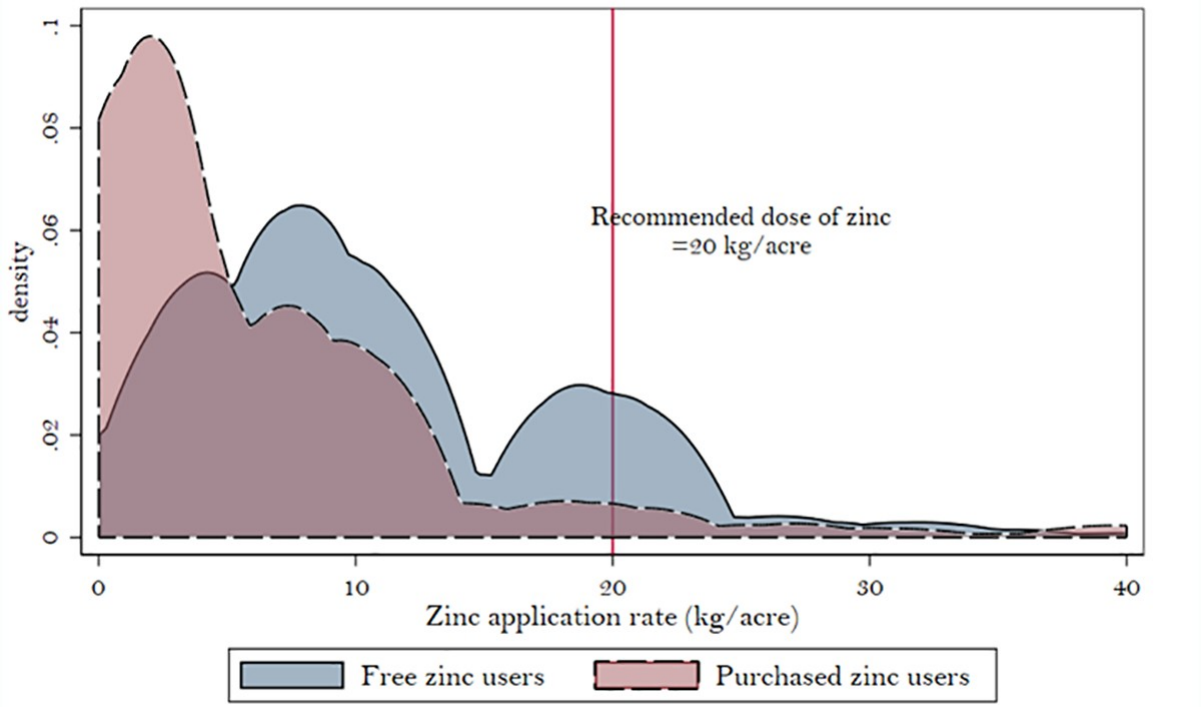
Zinc application rates for free zinc users vs. purchased zinc users. Source: Authors’ calculations.

Of our sample of 1,621 farmers, 720 had used zinc in the past year. Of these, 320 used free zinc provided by the state government, 390 purchased it from the market, and a small sample of 10 farmers used both free and purchased zinc. All three groups of farmers applied less than the recommended dose of zinc (Fig 3). As one would expect, the average application rate of zinc (kg/acre) is significantly lower for farmers who purchased zinc even after controlling for crop type, district, and soil zinc concentration (Table 4). Matched samples of free and market-purchase zinc users also show a similar difference in application rates (results available upon request). This implies that the gap between the recommended quantity and the actual application of zinc is higher when farmers pay the market price for it. The negative price elasticity of zinc could explain why farmers who buy zinc from the market apply it in smaller quantities.

**Table 4:** Determinants of zinc quantity.

Variables	Used Zinc in 2018–19
Used only purchased zinc = 1	−4.08***
	(-0.61)
Used both purchased and free zinc = 1	−3.50*
	(-2.01)
Cultivated in only Kharif = 1	4.48***
	(-1.42)
Cultivated in both Kharif and Rabi = 1	4.15***
	(-1.46)
Cultivated Paddy = 1	−1.88**
	(-0.75)
Zinc status of the village (ppm)	0.27
	(-0.23)
Farming experience (years)	0.16**
	(-0.07)
Farming experiencesq (years)	−0.002**
	(-0.001)
Tenant = 1	−.39
	(-0.67)
Irrigation = 1	1.92***
	(0.66)
Marginal farmer = 1	−.87
	(-0.84)
Small farmer = 1	0.56
	(-0.98)
Semi-medium farmer = 1	−.50
	(1.14)
Medium farmer = 1	0.05
	(1.39)
District Fixed Effects	Yes
R- squared	0.216
Number of observations	720

Note: Standard errors in parentheses

***, **, and * denote significance at 1%, 5%, and 10%, respectively. We also controlled for ownership of SHC, caste fixed effects, and gender and education status of the farmer.

If the potential users are given credible information about the new input and its properties, then the demand can become less sensitive to prices and price subsidies [41]. Thus, agriculture extension can change the elasticity of demand of inputs like zinc, and extension efforts and subsidies can have strong complementarities [37].

### Financial and administrative costs of delivering subsidized micronutrients

**Fig 4:**
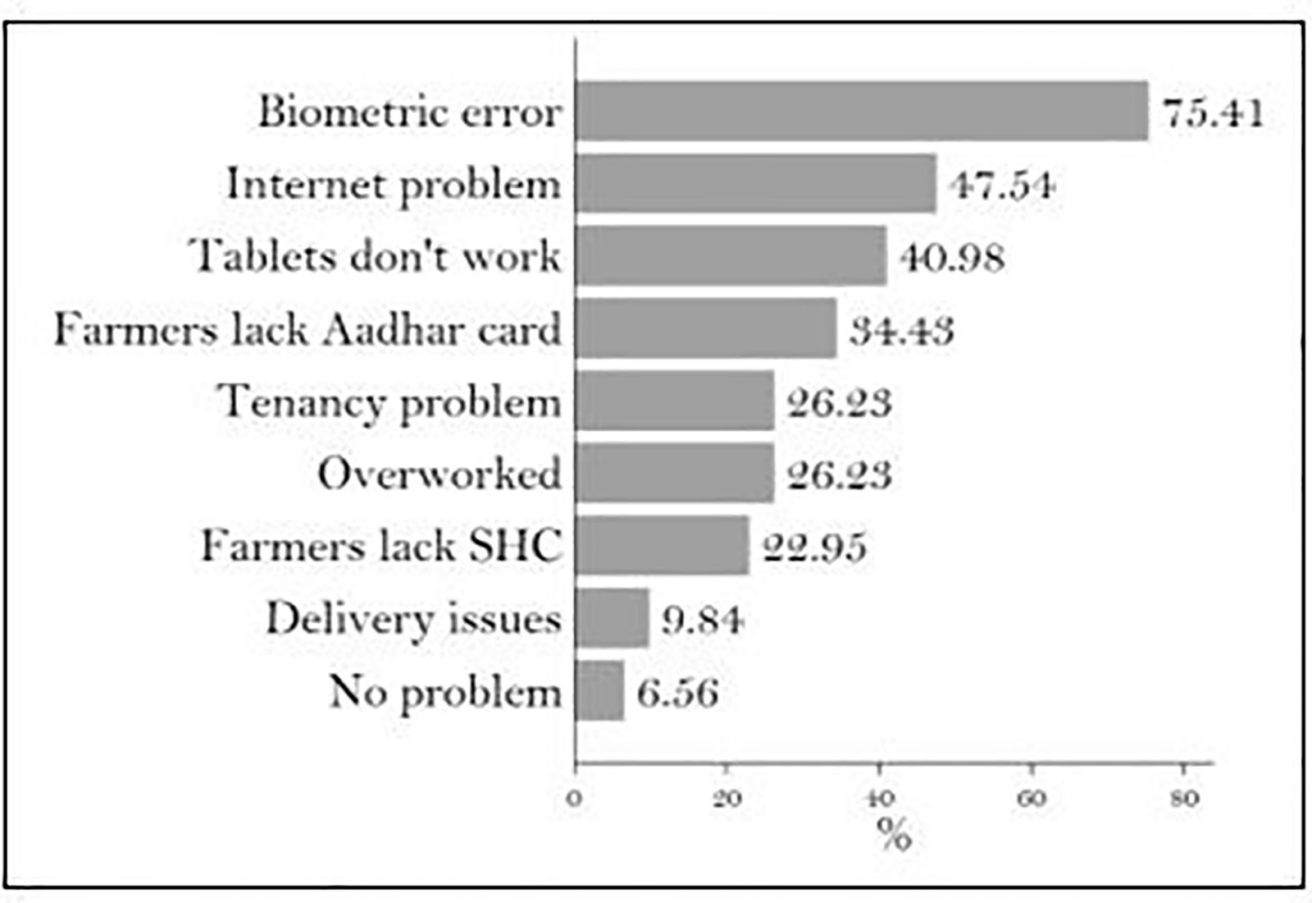
Concerns of extension staff. Source: Authors’ calculations.

The government of AP provides micronutrients free to farmers and shoulders the responsibility for their bulk purchase, transport, and retail distribution. Managing the procurement of 8,000 to 13,000 metric tons of zinc every year and distributing it to thousands of farmers and villages across the state adds an enormous amount of work to the already overburdened extension system. The responsibility of distributing free zinc and maintaining records of it leaves extension agents with less time for extension activities. One in four (26%) MPEOs in our sample reported that they were overburdened (Fig 4) and 37% of them had to work overtime for micronutrient distribution.

Currently, there are 4,095 MPEOs for nearly 8 million landholders in Andhra Pradesh [8]. This translates to an average of 2,000 farmers per MPEO, which is nearly three times the recommended ratio of one extension worker for 750 farmers [42].

**Fig 5:**
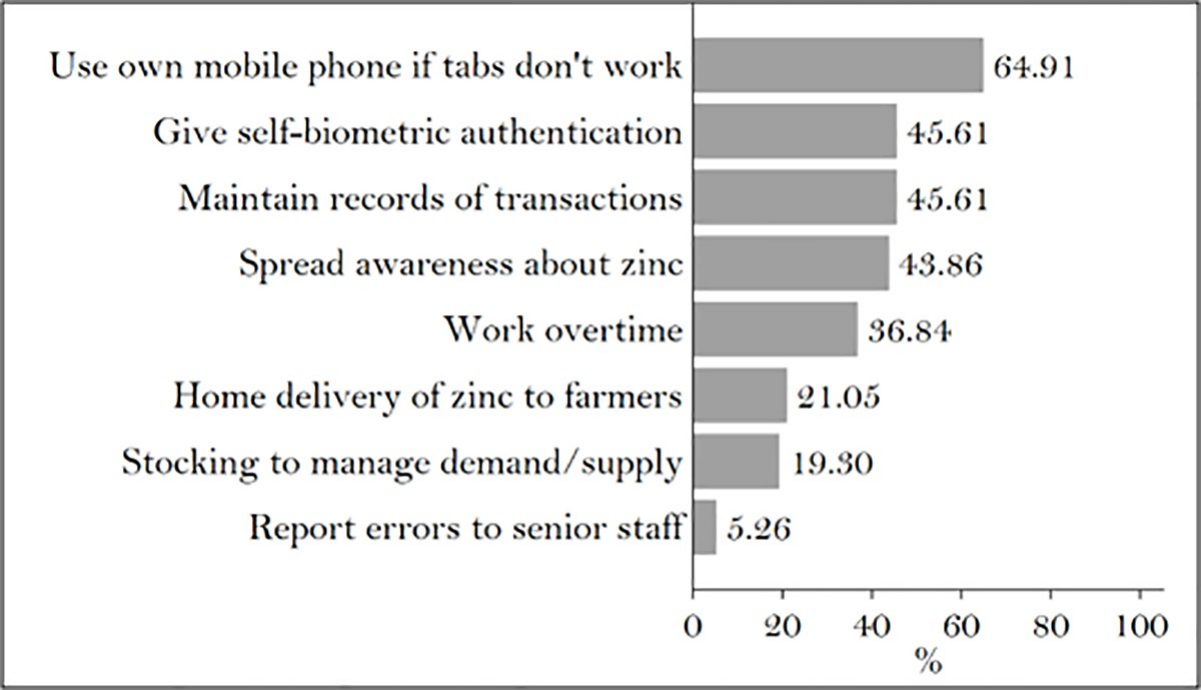
Troubleshooting methods used by extension staff. Source: Authors’ calculations.

MPEOs are required to conduct biometric verification of farmers and record micronutrient distribution data in the D-Krishi Android app. Errors in Aadhar authentication and poor connectivity in rural areas force MPEOs to record all the information on paper and enter it manually in the app at a later time. Instead of making the process easier and faster, MPEOs report that the app increases their workload (Fig 5).

In our analysis, we find that although the state can obtain zinc at low prices in bulk, the savings are offset by the high implementation costs discussed above. If the goal of the subsidy program was to generate demand, then this goal has already been met, as nearly 45% of farmers in our sample are already applying zinc. Additionally, most zinc users use nonsubsidized zinc, indicating that most farmers are willing to pay a positive price. In such a scenario, how can the implementation of this scheme be restructured, and what role can the private sector play in the state’s micronutrient space?

### Role of the private sector

In a competitive market, advertising and marketing by private stakeholders play a significant role in creating awareness about products and their usage. The role of public extension services is important, but effective coordination between public and private sector partners can intensify marketing efforts and improve public awareness. Much as agribusiness services are being encouraged through public-private partnerships in India [43], farmers’ lack of information about the usage and availability of zinc might be remedied by involving the private sector. In AP, there is a dual market for zinc: the public distribution of free zinc by the state agricultural department coexists with a commercial market for zinc products. In our sample, more than 50% (371 of 720) of farmers who had applied zinc to their fields had purchased it from private fertilizer shops. In such a dual-market setup, subsidized distribution by the government can crowd out commercial demand. A great amount of evidence comes from the largest input subsidy programs implemented in Sub Saharan Africa. Evidence suggests that for every kilogram of subsidized fertilizer, smart subsidies, with a targeted approach, crowded out 0.42–0.51 kg of commercial fertilizer in Kenya [44], 0.22 kg in Malawi (17) and 0.13 kg in Zambia [45], and reduced the probability of participation in commercial fertilizer market by 10–21% per 100 kg of subsidized fertilizer in Nigeria [46]. The crowding-out effect is stronger when the private sector has a large presence, as in AP [45]. Subsidies can help in crowding-in of commercial demand for inputs when there is uncertainty about the effectiveness of a new agricultural technology, and the private sector market for the technology is weak or nascent as in Uganda [13].

**Fig 6:**
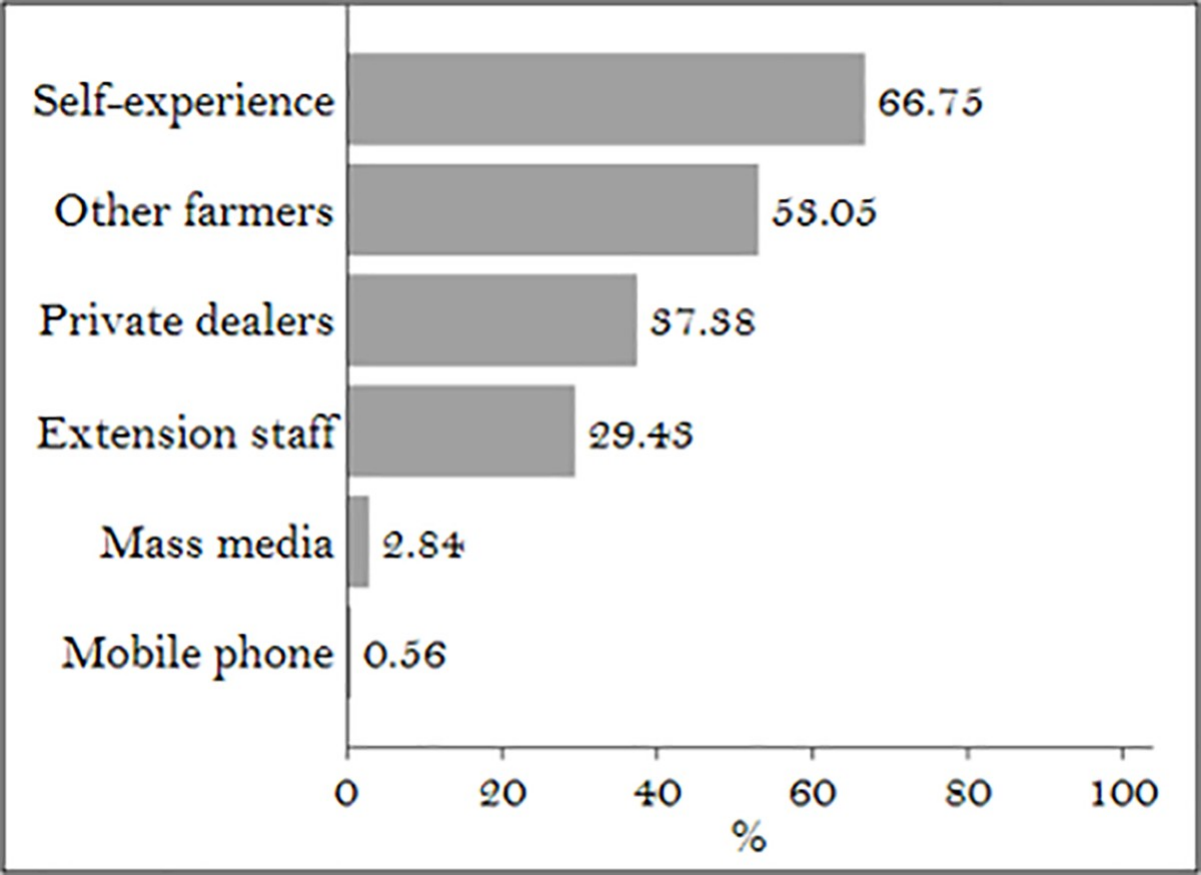
Major sources of information used by farmers. Source: Authors’ calculations.

In 2016, nearly 2,050 PACS reported to be operating in AP [47] were included in the scheme. At the same time, more than 8,000 active private dealers were reported to be operating in the state, out of which more than 7,000 were retailers [48]. On average, dealers and retailers are present within five or six kilometers from farmers’ homesteads and more often are closer points of contact for farmers than government extension agents. A local retailer can be a major source of information on crop inputs [49, 50] so that diffusion of knowledge becomes more likely in everyday interactions than in professionally facilitated meetings and activities [51]. Nearly 37% of the farmers in our sample reported private dealers as being a reliable source of information about agricultural practices (Fig 6). While the crowding out of commercial fertilizer use is beyond the scope of this paper, it can be argued that there is room to empower retailers and dealers with information that can be easily communicated to farmers. Since government extension agents are overstretched with managing multiple schemes, it important to assess the possibility and benefits of involving private sector partners in conducting awareness generation. One such avenue is on-farm demonstrations, a practice that the government of AP is encouraging as part of its extension activities [52].

In our discussions with private fertilizer companies in AP, we find that the provision of free zinc to farmers has eroded private markets, leading to lower zinc availability and choice for farmers. In 2018–19, only 7,922 metric tons of zinc had been supplied against the estimated demand of 30,000 metric tons. The role of the private sector to meet this demand and supply gap is essential, but without the right incentives, this gap will persist.

### Alternative fiscal and policy options

From our analysis of the current scheme, we propose a shift to DBT from the existing price subsidy regime for micronutrients in AP. In this setup, local dealers will be able to supply zinc and micronutrients from multiple brands, allowing farmers a choice of options at competitive prices. Farmers would be allowed to purchase any quantity and brand they prefer and pay these dealers for it. However, instead of receiving a discounted price paid as a subsidy, farmers will be reimbursed with a cash transfer up to a certain limit in their bank account after the purchase. This cash transfer can be fixed according to budget constraints. Private companies will also have the incentive to engage in greater market development and advertising to promote their products and improve product quality. With more choices, farmers can make informed decisions about what type and how much zinc to purchase. DBT of micronutrient subsidies will increase competition and incentivize fertilizer dealers to create greater awareness among farmers about the need for micronutrients in their soils.

### Identifying beneficiaries and subsidy rates

There are two methods of zinc application: basal and foliar. The average market price of Zinc Sulfate 21%, the most used formulation in AP, is INR 90 per kg; the price of Zinc EDTA is INR 450 per 0.5 kg. Assuming a farmer applies the recommended levels of zinc, basal application of zinc will cost INR 4,500 per ha and foliar application of zinc will cost INR 450 per ha in each season. The strategy of designing cash transfers depends on two conditions: identification and value. The first aspect involves deciding a suitable criterion to identify the beneficiary. For instance, using SHC data and farmer land records, cash transfers can identify farmers whose lands are deficient and subsidize zinc purchase only up to the recommended quantity. The value of this transfer can be indexed to the price of zinc to ensure that inflation does not erode its worth. The second aspect of DBT would involve deciding the amount of cash transfer. This will depend upon the budget constraints and the area that is targeted to be covered under the scheme. Presently, the state government obtains Zinc Sulfate 21% at INR 53 per kg and Zinc EDTA at INR 250 per kg (on average) which is 55% to 60% of their market price [28]. If the government wants to keep its budgetary outlay constant, it can provide a cash transfer of INR 53 per kg, requiring farmers to incur an effective cost of INR 37 per kg (market price minus subsidy) out of pocket for basal zinc application. This amount is not significantly different from the willingness to pay for zinc found previously. In other words, for every kg of zinc purchased, the government will reimburse the farmer roughly 60% of the price as a cash transfer as compared to the current subsidy rate of 100%.

In this scenario, the state can *subsidize* sales of nearly 14,000 metric tons of Zinc Sulfate 21%, which can cover approximately 3 lakh (300,000) ha of land in a single year (assuming the farmer applies recommended dosages). This amount will take care of the farmers who can purchase zinc; those who are severely credit constrained would need additional financial support in the form of improved credit products and facilities. Studies have shown that basal applications of zinc allow nutrients to be retained in the soil for three to five years after application [53]. Taking advantage of this finding, the government can implement the new policy starting with the most deficient regions first and cover the least deficient regions in subsequent years, in a phased manner, eventually covering the target area of 10 lakh (1 million) ha [29] in three years. AP is one of the few states in India with impressive penetration of Aadhar cards, SHCs, and banking provisions for farmers—all of which would be critical for the foundation of such a direct benefit transfer program.

Alternatively, if the state wants to continue making zinc free for farmers, but without the burden of procurement and distribution, then it would have to incur a cost of INR 90 per kg or INR 4,500 per ha in cash transfer through open market channels. This would mean that the farmers will be reimbursed entirely for the cost incurred to purchase zinc through a DBT after purchase. Assuming the same budget as before, the government can facilitate the sale of around 8,000 metric tons, which can cover around 1.6 (160,000) lakh hectares of land in a year, significantly lower than the scenario discussed previously.

## Discussion

The application of micronutrients is uncommon in India. In 2016–17, the latest year for which we have data on input use patterns, only 12.3% of all paddy growers applied any synthetic fertilizers, other than nitrogen, phosphorus, and potassium (NPK) [54]. This fraction was 14.3% for AP. The state government started offering a 50% subsidy on micronutrients in 2015 and free distribution began in 2016–17. In 2018–19, when we carried out our survey, 45% of the sample reported applying zinc to their crops. The free provision, therefore, has led to a notable jump in the use of zinc in the state within a few years. The sharp increase in the use of zinc shows that short-run targeted subsidies can help generate demand for agricultural inputs by promoting experiential and social learning. However, now that zinc is no longer a new or an unfamiliar input for most farmers in the state [54] and the commercial market for it is growing, there is very little justification to continue with the free public distribution.

There are three reasons why the state government should change the program design. First, free distribution can be justified only when the uptake of a product (or an input) generates large positive externalities and its demand falls precipitously even when only a small price is charged for it. Zinc use does not have any significant positive externalities, and our diagnostic survey shows that farmers are willing to pay a positive price for zinc.

Second, the distribution of free zinc is fiscally unsustainable. The price of zinc has risen in the international market and the state government is facing severe budget constraints in buying the required quantity, requiring it to be rationed. Rationing of an input or a product often leads to its inefficient and unfair allocation. In AP too, districts where zinc deficiency is the highest and its application will have the highest marginal returns do not receive more zinc, even as traditionally more prosperous irrigated districts receive more of the free zinc (see Fig 1).

Third, the state’s distribution of free zinc is crowding out the private sector. Through reference dependence or anchoring [55], the subsidy lowers the reservation price of zinc for many farmers to a level at which it is unviable for the private companies to sell. In this paper, we do not estimate the extent to which public provision crowds out commercial sales, but the effect may be large enough to reduce overall zinc use, especially in the richer irrigated areas of the state where the private sector is highly active [45]. In the beginning, free distribution helped increase the demand for Zinc, but now it is creating disincentives for private fertilizer companies, thereby, creating an artificial need to continue the government program.

### Policy recommendations and conclusion

This paper assesses the efficacy of the zinc subsidy program in AP and draws attention to the design and implementation problems. preventing the scheme from being effectively targeted. We show that the use of zinc, both paid and free is common in the state but several factors prohibit the efficient use of zinc subsidy in AP. We find that along with fiscal burden, the subsidy imposes a significant administrative burden on the state, tying up precious resources that could otherwise be spent on increasing information and awareness, that we find to be positively correlated with zinc use.

We recommend two changes to the subsidy policy: eliminating free zinc distribution and replacing it with a cash transfer, and channeling distribution through private dealers. Cost-sharing will reduce both the fiscal burden on the state government and the need to ration supplies. Channeling micronutrient subsidies through the commercial market will encourage competition, keep prices competitive, and improve quality. It also will free extension staff from distribution activities, allowing them to do their main task, which is bringing useful information to farmers.

The current system can be redesigned to promote a vibrant commercial sector for zinc by providing subsidies in the form of cash transfers to farmers in regions with soil deficiency. Farmers will then have the option to buy zinc of a brand and formulation of their choice from any one of the more than 8,000 registered fertilizer dealers in the state.

AP was the first state in India to create the necessary infrastructure and database needed to facilitate DBT of fertilizer subsidies [56]. All farmers in the state have Aadhar cards, and land records have been digitized, updated, and linked to Aadhar. In a few AP districts, the Aadhar cards have even been linked to SHC. All fertilizer retail shops in the state have point of sale (PoS) machines that can read biometric details, and the new fertilizer subsidy system already requires Aadhar-linked biometric verification of fertilizer sales [57]. The system created to implement the DBT of NPK fertilizers can be used for micronutrients as well.

Our survey data show that farmers who know about zinc and can detect symptoms of its deficiency are significantly more likely to use it in their fields. Farmers’ awareness about zinc is, in fact, the strongest predictor of its use (see Table 1). Providing more information on zinc application to farmers can change their price elasticity of demand and thus the impact of price subsidies [39]. The complementarity between subsidies and information can be large. State governments should, therefore, complement the DBT of zinc subsidies with a comprehensive training and awareness campaign on the benefits of zinc application.

The MPEOs can use the time saved from the distribution of zinc for these extension campaigns. The government should also engage private companies and their dealer networks in this campaign to increase its outreach. Lessons learned from our study in AP can be useful for rethinking and redesigning similar subsidy schemes to promote the use of macronutrients and micronutrients in other states of India and neighboring countries of South Asia, where alternatives to price subsidies are being considered.

## Supporting information

S1 Table Description of variables used in the study. (PDF)

S2 Table Determinants of use of free and purchased zinc. (PDF)

S1 File Farmer survey. (PDF)

S2 File Input dealer survey. (PDF)

S3 File Extension worker survey. (PDF)
