# Supplementary material for: Designing better input support programs: Lessons from zinc subsidies in Andhra Pradesh, India
Source: PLoS One. 2020 Dec 3;15(12):e0242161. doi: 10.1371/journal.pone.0242161 (PMC7714421; doi:10.1371/journal.pone.0242161)
Supplement: S1 Table — (PDF) [file pone.0242161.s001.pdf]

S1 Table. Description of variables used in the study

Variable	Description
Zinc Deficiency Identify	Dummy variable=1 if one can identify zinc deficiency in his/her land and 0 o/w
Land owned	Land owned in crop year 2018 in acres
Land ownedsq	Square of land owned above
Tenant	Dummy variable=1 if one was a tenant farmer in year 2018 and 0 o/w
Marginal farmer	Dummy variable=1 if (0.02<land owned <=1 ha) and 0 o/w
Small farmer	Dummy variable=1 if (1<land owned <=2 ha) and 0 o/w
Semi-medium farmer	Dummy variable=1 if (2<land owned <=4 ha) and 0 o/w
Medium farmer	Dummy variable=1 if (4<land owned <=10 ha) and 0 o/w
Scheduled Caste	Dummy variable=1 if one is Scheduled Caste and 0 o/w
Scheduled Tribe	Dummy variable=1 if one is Scheduled Tribe and 0 o/w
Other Backward Classes	Dummy variable=1 if one is from Other Backward Classes and 0 o/w
Educated	Dummy variable=1 if one has some formal education and 0 o/w
Used Zinc	Dummy variable=1 if used zinc in 2018 and 0o/w
Used Boron	Dummy variable=1 if used boron in 2018 and 0 o/w
Used Gypsum	Dummy variable=1 if used gypsum in 2018 and 0 o/w
Free Zinc	Dummy variable=1 if used free zinc in 2018 and 0 if not used zinc at all
Purchased Zinc	Dummy variable=1 if used purchased zinc in 2018 and 0 if not used zinc at all

Cultivated Paddy	Dummy variable=1 if one cultivated paddy as their major crop in 2018 and 0 o/w
Age	Age in years
Age squared	Age in years squared
Farming experience	Farming experience in years
Farming experiencesq	Farming experience in years squared
Male	Dummy variable=1 if respondent is male and 0 o/w
Family size	Number of family members in the household including self
Soil health card	Dummy variable=1 if one owns a SHC and 0 o/w
Knows the MPEO	Dummy variable=1 if one knows the MPEO and 0 o/w
Shop distance	Distance from shop in kilometers
Irrigation	Dummy variable=1 if irrigation was done in 2018 and 0 o/w
Asset index	Index of household asset position
District	Dummy variable=1 for each district and 0 o/w
MPEO	Dummy variable=1 for each MPEO and 0 o/w
Used only purchased zinc	Dummy variable=1 if used only purchased zinc in 2018 and 0 o/w
Used both purchased and free zinc	Dummy variable=1 if used both free and purchased zinc in 2018 and 0 o/w
Cultivated in only Kharif	Dummy variable=1 if cultivated only in kharif 2018 and 0 o/w
Cultivated in both Kharif and Rabi	Dummy variable=1 if cultivated in both kharif 2018 and rabi 2018-19 and 0 o/w
Zinc status of the village (ppm)	Level of zinc in village in parts per million
