# Supplementary material for: Designing better input support programs: Lessons from zinc subsidies in Andhra Pradesh, India
Source: PLoS One. 2020 Dec 3;15(12):e0242161. doi: 10.1371/journal.pone.0242161 (PMC7714421; doi:10.1371/journal.pone.0242161)
Supplement: S2 Table — (PDF) [file pone.0242161.s002.pdf]

S2 Table. Determinants of use of free and purchased zinc.

Characteristic	Used free zinc=1	Used mkt zinc=1	Difference in coefficients (free- buy)
Zinc deficiency identify=1	0.250***<br>(0.030)	0.284***<br>(0.032)	-0.034<br>(0.039)
Marginal=1	0.072*<br>(0.043)	-0.002<br>(0.045)	0.074<br>(0.056)
Small=1	0.096**<br>(0.046)	0.019<br>(0.051)	0.077<br>(0.063)
Semi-medium=1	0.142**<br>(0.054)	-0.054<br>(0.065)	0.196***<br>(0.071)
Medium=1	0.233***<br>(0.069)	-0.014<br>(0.098)	0.247**<br>(0.099)
Tenant=1	0.030<br>(0.033)	0.091***<br>(0.040)	-0.061<br>(0.041)
Scheduled class=1	0.011<br>(0.041)	0.064<br>(0.050)	-0.052<br>(0.058)
Scheduled Tribe=1	-0.143***<br>(0.055)	0.042<br>(0.050)	-0.185***<br>(0.071)
Other backward class=1	0.000<br>(0.032)	0.065**<br>(0.037)	-0.064<br>(0.044)
Educated=1	0.064***<br>(0.024)	0.050**<br>(0.026)	0.014<br>(0.032)
Used Boron=1	0.299***<br>(0.071)	0.271***<br>(0.075)	0.028<br>(0.089)
Used Gypsum=1	0.117***<br>(0.040)	0.022<br>(0.041)	0.095**<br>(0.048)
Farming experience	0.007**<br>(0.003)	0.006*<br>(0.003)	0.001<br>(0.004)
Farming experiencesq	-0.000**<br>(0.000)	-0.000<br>(0.000)	-0.000<br>(0.000)
Male=1	0.025<br>(0.036)	-0.059<br>(0.045)	0.084<br>(0.052)
Irrigation=1	-0.023<br>(0.030)	-0.029<br>(0.036)	0.005<br>(0.043)
Soil health card=1	0.096***<br>(0.050)	-0.088*<br>(0.057)	0.184***<br>(0.058)
Knows the MPEO=1	0.152***<br>(0.035)	-0.028<br>(0.038)	0.180***<br>(0.043)
Shop distance	-0.015***<br>(0.004)	-0.002<br>(0.004)	-0.013***<br>(0.005)
Cultivated Paddy=1	0.052*<br>(0.037)	0.112***<br>(0.036)	-0.060<br>(0.051)
District=1			
East Godavari	-0.208***<br>(0.059)	0.187***<br>(0.063)	-0.395***<br>(0.081)
Krishna	-0.246***<br>(0.048)	0.136***<br>(0.058)	-0.382***<br>(0.072)

Nellore	0.325***<br>(0.075)	0.268*<br>(0.093)	0.057<br>(0.083)
Srikakulam	0.058***<br>(0.058)	0.072<br>(0.047)	-0.013<br>(0.072)
West Godavari	-0.204***<br>(0.057)	0.297***<br>(0.074)	-0.500***<br>(0.098)
R square	0.37	0.20	-
Number of observations	1213	1283	-

Note: Standard errors in parentheses; ***, **, and * denote significance at 1%, 5%, and 10%, respectively. Standard errors are clustered around villages.
