# Supplementary material for: Designing better input support programs: Lessons from zinc subsidies in Andhra Pradesh, India
Source: PLoS One. 2020 Dec 3;15(12):e0242161. doi: 10.1371/journal.pone.0242161 (PMC7714421; doi:10.1371/journal.pone.0242161)
Supplement: S1 File — (PDF) [file pone.0242161.s003.pdf]

Farmer Diagnostic Survey

Q1. Enumerator ID

◯ 1 - ◯ 30

Q2. Is the respondent the major decision maker in the family on matters related to agriculture?

◯ (1) Yes

◯ (2) No

Consent form:

Greetings! I am ………….. from Neerman – an agency doing research in the state of Andhra Pradesh. You are being asked to participate in this survey being conducted by the International Food Policy Research Institute (IFPRI- a research institute engaged in agricultural research). We are doing this survey to find out about the farmer's knowledge of use of micronutrients and the Soil Health Card scheme. In this survey we will ask you questions on the challenges you are facing in using micronutrients (Zinc, born, gypsum etc), where are you getting information on them from and the cost you incur.

You have been selected randomly for this survey, which is being conducted across 6 selected districts of Andhra Pradesh among farmers who have cultivated in both the kharif and rabi seasons. Your participation in this survey will take about 30 to 40 minutes. If you have any questions about the survey, feel free to ask them at any point.

Before we start we would like you to know that: • You can participate if you want to. You have the right to withdraw from this survey at any point during the survey without giving reason. If you do not wish to participate at all, you have the alternative of quitting now. • By participating in this survey, you will not face any problems. We expect that the information obtained from this survey will be helpful in reforming the micronutrient schemes (might be useful to the Government of Andhra Pradesh and the Government of India). • Whether you participate in this survey or not, you will neither gain nor lose anything. • You have the right to confidentiality regarding the privacy of your personal information. • Whatever information that you are going to tell us, we won't tell anyone else. Only survey team will know about the responses you provide. • We are getting this information from several farmers like you, and we are going to prepare a report based on all this information, and no one farmer's name/village details will be shared.

Compensation: We are not going to provide you any compensation for participating in this survey. In case of any questions/conflicts, please contact Mr Amit Patil at 022-24021757, who will put you in touch with the research team.

Q3. Do you consent to participate in the survey?

◯ (1) Yes

◯ (2) No

If Q3=1, then ask questions from A.1 to S1.7 o/w skip to T.1.

A1. District ◯ district

A2. Mandal ◯ mandal

A3. Village ◯ village

A4. Respondent number

Please note that the Respondent ID for this respondent is ${respondent_id}

A5. Name of the respondent

A6. Father's name of the respondent

A7. Gender of the respondent

◯ (0) Male ◯ (1) Female ◯ (95) Others

A8. Age of the respondent

(in years)

A9. Contact number

(Enter your 10 digit mobile number) - If no number then write 0 ten times - Ex "0000000000"

A9.1. Alternative contact number

(Enter your 10 digit mobile number) - If no number then write 0 ten times - Ex "0000000000"

A10. Household caste

◯ (0) General caste ◯ (1) Scheduled Caste ◯ (2) Scheduled Tribe ◯ (3) Other Backward Class

◯ (95) Other

A10(TEXT). Specify other caste.

A11.1(TEXT). Specify your jaati.

A11. Jaati

◯ Brahman ◯ Kapu ◯ Kamma ◯ Komati ◯ Kshatriya ◯ Others ◯ Madiga ◯ Mala ◯ Balija ◯ Boya/Besta ◯ Chakali ◯ Devanga ◯ Dudekula ◯ Goundla ◯ Gavara ◯ Golla ◯ diga ◯ Jangam

◯ Kammara/ ◯ Vishwa ◯ Brahmana ◯ Kummari ◯ Kurma ◯ Munnurukapu ◯ angali ◯ Mutrasi ◯ Padmasali ◯ Telaga ◯ Uppara ◯ Waddera ◯ Velama ◯ Others

A11(TEXT). Specify other jaati.

A12. Household religion

◯ (0) Hindu ◯ (1) Muslim ◯ (2) Sikh ◯ (3) Christian ◯ (4) Buddhist ◯ (5) Jain ◯ (95) Other

A12(TEXT). Specify other religion.

A13. Marital status of the respondent

◯ (1) Single/Unmarried ◯ (2) Married ◯ (3) Divorced/Separated ◯ (4) Widowed ◯ (-98) Refused to answer

A14. Number of years of farming experience

(in years)

A15. How many family members are there in the respondent's family?

A16(a). What is the highest level of schooling acquired by the respondent?

◯ (0) No schooling

◯ (1) Primary (class 1-5) Completed

◯ (2) ME (class 6-7) Completed

◯ (3) High school (class 8-10) Completed

◯ (4) Intermediate (class 11-12) Completed

◯ (5) Graduate (Bachelors) Completed

◯ (6) Postgraduate (Masters) or higher Completed

◯ (7) Vocational training/Diploma

A16(b). (if A16(a) >=4) What was your main subject of specialization?

A16(c). What is the literacy level of the respondent?

◯ (0) Cannot read or write ◯ (1) Can write but cannot read ◯ (2) Can read but cannot write ◯ (3) Can read and write

A17. Is the respondent currently attending school or college?

◯ (1) Yes ◯ (2) No

Section B: Conversion units

B1. What is the principal unit you use for measuring land?

◯ Acre ◯ Guntha ◯ Cent ◯ Kuncham ◯ Hectare ◯ Decimal ◯ Mana ◯ Bharana ◯ Bigha ◯ Ankanam ◯ Ground ◯ Other

B1.1. Specify other land unit.

${land_unit_final} =value as selected in B1.

B2. How many ${land_unit_final} are there in 1 acre in your village?

(Put 99 if Don't know)

Section C: Land details

C1. Did you own any agricultural land in Kharif 2018? By own we mean land owned in your name or the combined household land, and not leased or rented.

◯ (1) Yes ◯ (2) No

C2. (if C1=1) How much agricultural land did you own in Kharif 2018?

(in ${land_unit_final})

C3. Did you own any agricultural land in Rabi 2018-19? By own we mean land owned in your name or the combined household land, and not leased or rented.

◯ (1) Yes ◯ (2) No

C4. (if C3=1) How much agricultural land did you own in Rabi 2018-19?

(in ${land_unit_final})

C5. In which of the two seasons did you cultivate any land?

(select all that apply)

☐ 1. Kharif 2018 ☐ 2. Rabi 2018-19

C6. (if C5=1) How much of the own land did you cultivate in Kharif 2018 (including Orchards)?

(in ${land_unit_final})

C7. (if C5=1) Did you cultivate any agricultural land as a tenant in Kharif 2018?

◯ (1) Yes ◯ (2) No

C8. (if C7=1) How much land did you cultivate as a tenant in Kharif 2018?

(in ${land_unit_final})

C9. (if C5=2) How much of the own land did you cultivate in Rabi 2018-19 (including Orchards) ?

(in ${land_unit_final})

C10. (if C5=2) Did you cultivate any agricultural land as a tenant in Rabi 2018-19?

◯ (1) Yes ◯ (2) No

C11. (if C10=1) How much land did you cultivate as a tenant in Rabi 2018-19?

(in ${land_unit_final})

Repeat the following questions for all seasons selected in C5. If C5=1, then ${seasonnumber}=Kharif 2018, if C5=2, then ${seasonnumber}= Rabi 2018-19.

SECTION: ${seasonnumber}

D1. Which of the following was the primary crop you cultivated in ${seasonnumber}? (By primary crop, we mean the crop for which there was MAXIMUM AREA UNDER CULTIVATION)

◯ Paddy ◯ Wheat ◯ Sugarcane ◯ Maize

◯ Pulses (dal, moong, rajma, channa, chole, etc.)

◯ Oilseeds (mustard, sesame, groundnut, castor, linseed, etc.)

◯ Vegetables ◯ Other

D1.1. Specify other primary crop.

D2. Which of the following was the secondary crop you cultivated in ${seasonnumber} ? (By secondary crop, we mean the crop for which there was 2nd largest area under cultivation on the remaining land

◯ Paddy ◯ Wheat ◯ Sugarcane ◯ Maize

◯ Pulses (dal, moong, rajma, channa, chole, etc.)

◯ Oilseeds (mustard, sesame, groundnut, castor, linseed, etc.)

◯ Vegetables ◯ Other ◯ None

D2.1. Specify other secondary crop.

${prim_cropname} = crop as selected in D1.

We are now going to ask you questions about crop ${prim_cropname} that you cultivated in ${seasonnumber}

E1n1. How much area did you cultivate in ${seasonnumber} under ${prim_cropname}?

(in ${land_unit_final}; including cultivation on owned land and leased land)

E2n2. How much area do you plan to cultivate under ${prim_cropname} next year in the same season?

E3n3. In which month was ${prim_cropname} sown in ${seasonnumber} ?

◯ (1) April, 2018 ◯ (2) May, 2018 ◯ (3) June, 2018 ◯ (4) July, 2018 ◯ (5) August, 2018

◯ (6) September, 2018 ◯ (7) October, 2018 ◯ (8) November, 2018 ◯ (9) December, 2018

◯ (10) January, 2019 ◯ (11) February, 2019 ◯ (12) March, 2019 ◯ (95) Don't know/remember

E4n4. Have you collected the harvest for ${prim_cropname} for the season ${seasonnumber} ?

◯ (1) Yes ◯ (2) No

E4n5. (if E4n4=1) When was ${prim_cropname} harvested?

◯ (1) March, 2018 ◯ (2) April, 2018 ◯ (3) May, 2018 ◯ (4) June, 2018 ◯ (5) July, 2018

◯ (6) August, 2018 ◯ (7) September, 2018 ◯ (8) October, 2018 ◯ (9) November, 2018

◯ (10) December, 2018 ◯ (11) January, 2019 ◯ (12) February, 2019 ◯ (13) March, 2019

◯ (95) Don't know/remember

E4n6. (if E4n4=2) When do you plan to harvest ${prim_cropname}?

◯ (1) March, 2018 ◯ (2) April, 2018 ◯ (3) May, 2018 ◯ (4) June, 2018 ◯ (5) July, 2018

◯ (6) August, 2018 ◯ (7) September, 2018 ◯ (8) October, 2018 ◯ (9) November, 2018

◯ (10) December, 2018 ◯ (11) January, 2019 ◯ (12) February, 2019 ◯ (13) March, 2019

◯ (14) April, 2019 ◯ (15) May, 2019 ◯ (16) June, 2019 ◯ (17) Harvest lost

◯ (-99) Don't know/remember

Section E1: Zinc

E1. Did you apply Zinc in cultivating ${prim_cropname} in ${seasonnumber}?

◯ (1) Yes ◯ (2) No

E2. (if E1=2) Why did you not apply any Zinc in ${seasonnumber} for cultivating ${prim_cropname}?

(select all that apply)

☐ (1) Not needed on my land

☐ (2) Not interested

☐ (3) Market/PACS too far

☐ (4) Not available in the nearest source

☐ (5) Not effective on my land from past experience

☐ (6) Did not have money to buy Zinc

☐ (7) Don’t have money to bear application costs

☐ (8) Unsure about its impact

☐ (9) Did not know about Zinc fertilizer

☐ (10) Did not know of positive impact of Zinc

☐ (11) Rented land

☐ (12) Did not know about subsidy scheme of Zinc fertilizer

☐ (95) Other reason

E2.1. Specify other reason.

Ask E3- E22.1 if E1=1:-

E3. What were the reasons for applying Zinc to your land for ${prim_cropname} in ${seasonnumber}?

(select all that apply)

☐ (1) Has a positive impact on yield

☐ (2) Friends or other farmers encouraged to add

☐ (3) Because it is free

☐ (4) MPEO encouraged to add Zinc

☐ (5) Heard about its positive impact from other farmers

☐ (6) Got to know about Zinc deficiency from Soil Health Card

☐ (7) Got to know about Zinc deficiency through soil testing

☐ (95) Other reason

E3.1. Specify other reason.

E5n1. How did you get Zinc in ${seasonnumber} to cultivate ${prim_cropname}?

☐ (1) Obtained Free Zinc ☐ (2) Purchased Zinc

E5. (if E5n1=1) What was your major source of obtaining Free Zinc in ${seasonnumber} for ${prim_cropname}?

◯ Primary Agricultural Cooperative Society (PACS)

◯ Mandal Office

◯ Friend/relative (Borrowed)

◯ Previous year's stock (Own Zinc)

◯ Other source

E5.1. Specify other source of obtaining Free Zinc.

${freezinc_sourcename}= value as selected in E5.

E6. (if E5n1=1) How much free Zinc did you obtain from ${freezinc_sourcename}?

(in kilograms) (Put 99 if Don’t know)

E8. (if E5n1=2) What was your major source of buying Zinc in ${seasonnumber} for ${prim_cropname}?

◯ Primary Agricultural Cooperative Society (PACS)

◯ Mandal Office

◯ Private Fertilizer Shops

◯ Other source

E8.1. Specify other source of buying Zinc.

${buyzinc_sourcename} = value as selected in E8.

E9. (if E5n1=2) How much Zinc did you buy from ${buyzinc_sourcename}?

(in kilograms) (Put 99 if Don’t know)

E10. (if E5n1=2) What was the total cost of buying Zinc from ${buyzinc_sourcename}?

(in Rupees) (Put 99 if don’t know)

E11. What was the method of application that you used for Zinc?

(select all that apply)

☐ (1) Basal Application ☐ (2) Foliar Application

E13. How much total Zinc did you apply to ${prim_cropname} in ${seasonnumber}?

(in kilograms) Put 99 if Don’t know

${area_current}= value as in E1n1

E14. Out of the ${area_current} ${land_unit_final} of land cultivated for ${prim_cropname}, on how much land did you apply Zinc?

(in ${land_unit_final})

E15. Did you hire any labour to apply Zinc to ${prim_cropname} in ${seasonnumber}?

◯ (1) Yes ◯ (2) No

E16. (if E15=1) How much total cost of labour did you incur for application of Zinc?

(in Rupees) Put 99 if don’t know

E16.1. (if E15=1) Was this labour hired just for applying Zinc or for applying all inputs together?

◯ (1) Yes, just for Zinc ◯ (2) No, for all inputs together

E17. Have you rented or bought any devices / tools / machines to apply Zinc for ${prim_cropname} in ${seasonnumber}?

☐ (1) Yes, bought ☐ (2) Yes, hired ☐ (3) No

E18. (if E17=1 or E17=2) How much was total cost of those machinery/tools etc for applying Zinc?

(in Rupees) Put 99 if don't know

E19. Did you observe any positive impact of applying Zinc on ${prim_cropname} yield in ${seasonnumber} as compared to last year?

◯ (1) Yes ◯ (2) No

E20. (if E19=1) Which of the following positive impacts of applying Zinc to ${prim_cropname} did you observe in ${seasonnumber} as compared to last year?

(select all that apply)

☐ (1) Improvement in yield quantity

☐ (2) Improvemnet in yield quality

☐ (3) Increased price realized in the market

☐ (4) Reduction in fertilizer usage

☐ (5) Improvement in soil quality

☐ (95) Other

E20.1. Specify other positive impact that you observed.

E21. Did you observe any negative impact of applying Zinc on ${prim_cropname} yield in ${seasonnumber} as compared to last year?

◯ (1) Yes ◯ (2) No

E22. (if E21=1) Which of the following negative impacts of applying Zinc to ${prim_cropname} did you observe in ${seasonnumber} as compared to last year?

(select all that apply)

☐ (1) Reduction in yield quantity

☐ (2) Deterioration of yield quality

☐ (3) Decreased price realized in the market

☐ (4) Increase in fertilizer usage

☐ (5) Deterioration in Soil quality

☐ (95) Other

E22.1. Specify other negative impact that you observed.

Section E2: Boron

E23. Did you apply Boron in cultivating ${prim_cropname} in ${seasonnumber}?

◯ (1) Yes ◯ (2) No

E24. If E23=2) (Why did you not apply any Boron in ${seasonnumber} for cultivating ${prim_cropname} ?

(select all that apply)

☐ (1) Not needed on my land

☐ (2) Not interested

☐ (3) Market/PACS too far

☐ (4) Not available in the nearest source

☐ (5) Not effective on my land from past experience

☐ (6) Did not have money to buy Boron

☐ (7) Don’t have money to bear application costs

☐ (8) Unsure about its impact

☐ (9) Did not know about boron fertilizer

☐ (10) Did not know of positive impact of boron

☐ (11) Rented land

☐ (12) Did not know about subsidy scheme of boron fertilizer

☐ (95) Other reason

E24.1. Specify other reason.

If E23=1, then ask E25-E44.1

E25. What were the reasons for applying Boron to your land for ${prim_cropname} in ${seasonnumber}?

(select all that apply)

☐ (1) Has a postive impact on yield

☐ (2) Friends or other farmers encouraged to add

☐ (3) Because it is free

☐ (4) MPEO encouraged to add boron

☐ (5) Heard about its positive impact from other farmers

☐ (6) Got to know about boron deficiency from Soil Health Card

☐ (7) Got to know about boron deficiency through Soil testing

☐ (95) Other reason

E25.1. Specify other reason.

E26. Do you remember the year in which you started using Boron?

◯ (1) Yes ◯ (2) No

E26.1. (if E26=1) In which year did you start using Boron? (Please enter year greater than 1950 and less than 2020.)

E27n1. How did you get Boron in ${seasonnumber} to cultivate ${prim_cropname}?

☐ (1) Obtained Free Boron ☐ (2) Obtained Boron at market price (not subsidized)

E27. (if E27n1=1) What was your major source of obtaining Free Boron in ${seasonnumber} for ${prim_cropname}?

◯ Primary Agricultural Cooperative Society (PACS)

◯ Mandal Office

◯ Friend/relative (Borrowed)

◯ Previous year's stock (Own Boron)

◯ Other source

E27.1. Specify other source of obtaining Free Boron.

${freeboron_sourcename} =value as in E27

E28. (if E27n1=1) How much free Boron did you obtain from ${freeboron_sourcename}?

(in kilograms) Put 99 if Don't know

E30. (if E27n1=2) What was your major source of buying Boron in ${seasonnumber} for ${prim_cropname}?

◯ Primary Agricultural Cooperative Society (PACS)

◯ Mandal Office

◯ Private Fertilizer Shops

◯ Other source

E30.1. Specify other source of buying Boron.

${buyboron_sourcename}= value in E30

E31. How much Boron did you buy from ${buyboron_sourcename}?

(in kilograms) Put 99 if Don't know

E32. What was the total cost of buying Boron from ${buyboron_sourcename}?

(in Rupees) Put 99 if don't know

E33. What was the method of application that you used for Boron?

(select all that apply)

☐ (1) Basal Application ☐ (2) Foliar Application

E35. How much total Boron did you apply to ${prim_cropname} in ${seasonnumber} ?

(in kilograms) Put 99 if Don't know

E36. Out of the ${area_current} ${land_unit_final} of land cultivated for ${prim_cropname} , on how much land did you apply Boron?

(in ${land_unit_final})

E37. Did you hire any labour to apply Boron to ${prim_cropname} in ${seasonnumber}?

◯ (1) Yes ◯ (2) No

E38. (if E37=1) How much total cost of labour did you incur for application of Boron?

(in Rupees) Put 99 if don't know

E38.1. (if E37=1) Was this labour hired just for applying Boron or for applying all inputs together?

◯ (1) Yes, just for Boron ◯ (2) No, for all inputs together

E39. Have you rented or bought any devices / tools / machines to apply Boron for ${prim_cropname} in ${seasonnumber}?

☐ (1) Yes, bought ☐ (2) Yes, hired ☐ (3) No

E40. (if E39=1 or E39=2) How much was total cost of those machinery/tools etc for applying Boron?

(in Rupees) Put 99 if don't know

E41. Did you observe any positive impact of applying boron on ${prim_cropname} yield in ${seasonnumber} as compared to last year?

◯ (1) Yes ◯ (2) No

E42. (if E41=1) Which of the following positive impacts of applying boron to ${prim_cropname} did you observe in ${seasonnumber} as compared to last year?

(select all that apply)

☐ (1) Improvement in yield quantity

☐ (2) Improvemnet in yield quality

☐ (3) Increased price realized in the market

☐ (4) Reduction in fertilizer usage

☐ (5) Improvement in soil quality

☐ (95) Other

E42.1. Specify other positive impact that you observed.

E43. Did you observe any negative impact of applying boron on ${prim_cropname} yield in ${seasonnumber} as compared to last year?

◯ (1) Yes ◯ (2) No

E44. (if E43=1) Which of the following negative impacts of applying boron to ${prim_cropname} did you observe in ${seasonnumber} as compared to last year?

(select all that apply)

☐ (1) Reduction in yield quantity

☐ (2) Deterioration of yield quality

☐ (3) Decreased price realized in the market

☐ (4) Increase in fertilizer usage

☐ (5) Deterioration in Soil quality

☐ (95) Other

E44.1. Specify other negative impact that you observed.

Section E3: Gypsum

E45. Did you apply Gypsum in cultivating ${prim_cropname} in ${seasonnumber}?

◯ (1) Yes ◯ (2) No

E46. (if E45=2) Why did you not apply any Gypsum in ${seasonnumber} for cultivating ${prim_cropname} ?

(select all that apply)

☐ (1) Not needed on my land

☐ (2) Not interested

☐ (3) Market/PACS too far

☐ (4) Not available in the nearest source

☐ (5) Not effective on my land from past experience

☐ (6) Did not have money to buy Gypsum

☐ (7) Don't have money to bear application costs

☐ (8) Unsure about its impact

☐ (9) Did not know about gypsum fertilizer

☐ (10) Did not know of positive impact of gypsum

☐ (11) Rented land

☐ (12) Did not know about subsidy scheme of gypsum fertilizer

☐ (95) Other reason

E46.1. Specify other reason.

If E45=1, then ask E47-E66.1

E47. What were the reasons for applying Gypsum to your land for ${prim_cropname} in ${seasonnumber}?

(select all that apply)

☐ (1) Has a postive impact on yield

☐ (2) Friends or other farmers encouraged to add

☐ (3) Because it is free

☐ (4) MPEO encouraged to add gypsum

☐ (5) Heard about its positive impact from other farmers

☐ (6) Got to know about gypsum deficiency from Soil Health Card

☐ (7) Got to know about gypsum deficiency through soil testing

☐ (95) Other reason

E47.1. Specify other reason.

E48. Do you remember the year in which you started using Gypsum?

◯ (1) Yes ◯ (2) No

E48.1. (if E48=1) In which year did you start using Gypsum? Please enter year greater than 1950 and less than 2020.

E49n1. How did you get Gypsum in ${seasonnumber} to cultivate ${prim_cropname}?

☐ (1) Obtained Free Gypsum ☐ (2) Obtained Gypsum at market price (not subsidized)

E49. (if E48n1=1) What was your major source of obtaining Free Gypsum in ${seasonnumber} for ${prim_cropname}?

◯ Primary Agricultural Cooperative Society (PACS)

◯ Mandal Office

◯ Friend/relative (Borrowed)

◯ Previous year's stock (Own Gypsum)

◯ Other source

E49.1. Specify other source of obtaining Free Gypsum.

${freegypsum_sourcename} = value as in E49

E50. How much free Gypsum did you obtain from ${freegypsum_sourcename}?

(in kilograms) Put 99 if Don’t know

E52. (if E48n1=2) What was your major source of buying Gypsum in ${seasonnumber} for ${prim_cropname} ?

◯ Primary Agricultural Cooperative Society (PACS)

◯ Mandal Office

◯ Private Fertilizer Shops

◯ Other source

E52.1. Specify other source of buying Gypsum.

${buygypsum_sourcename} – value as in E52.

E53. How much Gypsum did you buy from ${buygypsum_sourcename}?

(in kilograms) Put 99 if Don’t know

E54. What was the total cost of buying Gypsum from ${buyzinc_sourcename}?

(in Rupees) Put 99 if don’t know

E55. What was the method of application that you used for Gypsum?

(select all that apply)

☐ (1) Basal Application ☐ (2) Foliar Application

E57. How much total Gypsum did you apply to ${prim_cropname} in ${seasonnumber} ?

(in kilograms) Put 99 if Don’t know

E58. Out of the ${area_current} ${land_unit_final} of land cultivated for ${prim_cropname} , on how much land did you apply Gypsum?

(in ${land_unit_final})

E59. Did you hire any labour to apply Gypsum to ${prim_cropname} in ${seasonnumber}?

◯ (1) Yes ◯ (2) No

E60. (if E59=1) How much total cost of labour did you incur for application of Gypsum?

(in Rupees) Put 99 if don't know

E38.1. (if E59=1) Was this labour hired just for applying Gypsum or for applying all inputs together?

◯ (1) Yes, just for Gypsum ◯ (2) No, for all inputs together

E61. Have you rented or bought any devices / tools / machines to apply gypsum for ${prim_cropname} in ${seasonnumber}?

☐ (1) Yes, bought ☐ (2) Yes, hired ☐ (3) No

E62. (if E61=1 or E61=2)How much was total cost of those machinery/tools etc for applying Gypsum ?

(in Rupees) Put 99 if don't know

E63. Did you observe any positive impact of applying gypsum on ${prim_cropname} yield in ${seasonnumber} as compared to last year?

◯ (1) Yes ◯ (2) No

E64. (if E63=1) Which of the following positive impacts of applying gypsum to ${prim_cropname} did you observe in ${seasonnumber} as compared to last year?

(select all that apply)

☐ (1) Improvement in yield quantity

☐ (2) Improvement in yield quality

☐ (3) Increased price realized in the market

☐ (4) Reduction in fertilizer usage

☐ (5) Improvement in soil quality

☐ (95) Other

E64.1. Specify other positive impact that you observed.

E65. Did you observe any negative impact of applying gypsum on ${prim_cropname} yield in ${seasonnumber} as compared to last year?

◯ (1) Yes ◯ (2) No

E66. (if E65=1) Which of the following negative impacts of applying gypsum to ${prim_cropname} did you observe in ${seasonnumber} as compared to last year?

(select all that apply)

☐ (1) Reduction in yield quantity

☐ (2) Deterioration of yield quality

☐ (3) Decreased price realized in the market

☐ (4) Increase in fertilizer usage

☐ (5) Deterioration in Soil quality

☐ (95) Other

E66.1. Specify other negative impact that you observed.

Section F: Fertilizer

F1. Did you use any fertilizer for cultivating ${prim_cropname} in ${seasonnumber} ?

◯ (1) Yes ◯ (2) No

F2. (if F1=2) Why did you not use any fertilizer in ${seasonnumber} for ${prim_cropname}?

(select all that apply)

☐ (1) I grow organic

☐ (2) Can't afford fertilizer

☐ (3) Manure is enough for what I do

☐ (4) Fertilizer not available in my area

☐ (5) Can't bear application costs of fertilizer

☐ (95) Other reason

F2.1. Specify other reason for not using fertilizer.

If F1=1, then ask F3- F13.

F3. Which of the following fertilizers did you use to cultivate ${prim_cropname} in ${seasonnumber}? (Read out the options to the farmer)

(select all that apply)

☐ Urea

☐ DAP (Di-ammonium Phosphate)

☐ MOP (Muriate of Potash)

☐ SSP (Single Super Phosphate)

☐ Complex

☐ None of the above

Repeat the questions F4-F13 for all fertilizers selected in F3. ${fertnumber}= value selected in F3.

We are now going to ask you questions about fertilizer: ${fertnumber}

F4. How much ${fertnumber} did you apply for cultivating ${prim_cropname} in ${seasonnumber} ?

(in kilograms) Put 99 if Don't know

F6. What was the method of application that was mostly used by you?

◯ (1) Basal Application ◯ (2) Foliar Application

F7. Did you hire any labour to apply ${fertnumber} to ${prim_cropname} in ${seasonnumber}?

◯ (1) Yes ◯ (2) No

F8. (if F7=1) How much total cost of labour did you incur for application of ${fertnumber} ?

(in Rupees) Put 99 if don't know

F8.1. (if F7=1) Was this labour hired just for applying ${fertnumber} or for applying all inputs together?

◯ (1) Yes, just for ${fertnumber} ◯ (2) No, for all inputs together

F9. Have you rented or bought any devices / tools / machines to apply ${fertnumber} for ${prim_cropname} in ${seasonnumber}?

☐ (1) Yes, bought ☐ (2) Yes, hired ☐ (3) No

F10. (if F9=1 or F9=2) How much was total cost of those machinery/tools etc for applying ${fertnumber} ?

(in Rupees) Put 99 if don’t know

F11. What was the major source of obtaining ${fertnumber} for ${prim_cropname} in ${seasonnumber}?

◯ (1) Primary Agricultural Cooperative Society (PACS)

◯ (2) Mandal Office

◯ (3) Private Fertilizer Shops

◯ (4) Friend/relative (Borrowed)

◯ (5) Previous year's stock (Own Fertilizer)

◯ (95) Other source

F11.1. Specify other source of obtaining ${fertnumber}.

${fertsourcenumber}= value as in F11.

F12. How much ${fertnumber} did you buy from ${fertsourcenumber} ?

(in kilograms) Put 99 if Don’t know

F13. What was the total cost of buying ${fertnumber} from ${fertsourcenumber}?

(in Rupees) Put 99 if don’t know

Section G: Seeds

We are now going to ask you questions about the Seed used for cultivating ${prim_cropname} in ${seasonnumber}

G1. How much total seed of ${prim_cropname} did you use for cultivation in ${seasonnumber}?

(in kilograms or number) Put 99 if Don’t know

G2. What were the sources from which you obtained seed for ${prim_cropname}?

(select all that apply)

☐ Primary Agricultural Cooperative Society (PACS)

☐ Mandal Office

☐ Private Fertilizer Shops

☐ Borrowed from friend/relative

☐ Previous year's stock (own seed)

☐ Other source

G2.1. Specify other source of seed.

G3. How much seed of ${prim_cropname} in ${seasonnumber} did you obtain from the following:-

G3a. Primary Agricultural Cooperative Society (PACS) (in kilograms) Put 99 if Don't know

G3b. Mandal Office (in kilograms) Put 99 if Don't know

G3.c. Fertilizer Shops (in kilograms) Put 99 if Don't know

G3.d. Friends/Relatives (in kilograms) Put 99 if Don't know

G3e. Previous year stocks (in kilograms) Put 99 if Don't know

G3f. ${seed_source_other} (in kilograms) Put 99 if Don't know

G4. How much was the total cost of buying seed for ${prim_cropname} ?

(in Rupees) Put 99 if don't know

Section H: Other inputs

We are now going to ask you questions about the various other inputs used for cultivating ${prim_cropname} in ${seasonnumber}.

H1. Did you use any manure for cultivating ${prim_cropname} in ${seasonnumber} ?

◯ (1) Yes ◯ (2) No

H2. (if H1=1) How much total amount of manure did you use?

Put 99 if Don't know

H2.1. (if H1=1) What was your unit of measurement for manure?

◯ Tonnes ◯ Quintals ◯ Kilograms ◯ Litres ◯ Grams ◯ Other

H3. Did you use any other organic matter for cultivating ${prim_cropname} in ${seasonnumber} ?

◯ (1) Yes ◯ (2) No

H4. (if H3=1) How much total amount of Organic matter did you use ?

Put 99 if Don't know

H4.1. (if H3=1) What was your unit of measurement for organic matter?

◯ Tonnes ◯ Quintals ◯ Kilograms ◯ Litres ◯ Grams ◯ Other

H5. Which of the following is the primary source of irrigation that you used to cultivate ${prim_cropname} in ${seasonnumber}?

◯ (1) Rainfed ◯ (2) River ◯ (3) Tubewell (government) ◯ (4) Electric pump

◯ (5) Diesel pump ◯ (6) Pond ◯ (7) Canal ◯ (8) Well ◯ (95) Others

H5.1. Specify other source of irrigation.

H6. How much area was irrigated out of the ${area_current} ${land_unit_final} cultivated for ${prim_cropname} in ${seasonnumber} ?

(in ${land_unit_final})

H6.1. Did you face any cost of irrigation for cultivating ${prim_cropname} in ${seasonnumber}?

◯ (1) Yes ◯ (2) No

H7. (if H6.1=1) What was the total cost of irrigation for ${prim_cropname} in ${seasonnumber} ?

(in Rupees) Put 99 if Don't know

H8. Did you use any machine to cultivate ${prim_cropname} in ${seasonnumber} ?

◯ (1) Yes ◯ (2) No

H8n1. (if H8=1) Which of the following machinery did you use to cultivate ${prim_cropname} in ${seasonnumber}?

Read out all and ask to select (Please read out the names of the machines to the farmer)

Laser Guided Land Leveller ◯ (1) Yes ◯ (2) No

Axial flow thresher ◯ (1) Yes ◯ (2) No

Seed drill ◯ (1) Yes ◯ (2) No

Reaper ◯ (1) Yes ◯ (2) No

Tractor ◯ (1) Yes ◯ (2) No

Mechanical Rice Transplanter ◯ (1) Yes ◯ (2) No

Power Tiller ◯ (1) Yes ◯ (2) No

Combined harvester ◯ (1) Yes ◯ (2) No

Disc Harrow ◯ (1) Yes ◯ (2) No

Disc Plough ◯ (1) Yes ◯ (2) No

Mb Plough ◯ (1) Yes ◯ (2) No

Rotavator ◯ (1) Yes ◯ (2) No

Knapsack Sprayer ◯ (1) Yes ◯ (2) No

H9. (if H8=2) Why did you not use any machine in ${seasonnumber} to cultivate ${prim_cropname} ?

(select all that apply)

☐ (1) Can't afford to purchase a machine

☐ (2) Rental rate of the machine is higher than labour rate

☐ (3) Not available easily in the market or market is far away

☐ (4) Don't know how to operate a machine

☐ (5) Not aware about availability of a machine in the village

☐ (95) Other

H9.1. Specify other reason for not using a machine.

H10. Which of the following inputs did you use for cultivating ${prim_cropname} in ${seasonnumber} ?

(select all that apply)

☐ (1) Pesticides ☐ (2) Insecticides ☐ (3) Herbicides ☐ (4) Weedicides ☐ (98) None of the above

Section I: Harvest

We are now going to ask you questions related to Yield and Harvest of ${prim_cropname} in ${seasonnumber}

I1. How much was the total harvest of ${prim_cropname} in ${seasonnumber} ?

Put 99 if Don't know

I1.1. What was your unit of measurement for harvest of ${prim_cropname} ?

◯ Tonnes ◯ Quintals ◯ Kilograms ◯ Litres ◯ Grams ◯ Bags ◯ Other

I1.2. Specify other unit.

${harvesting_unit_final}= value as in I1.1

I1.3. How many kilograms are there in 1 ${harvesting_unit_final}

How much of the harvest was allocated as follows:-

I2.a.1. Given to landowner if land not owned?

(in ${harvesting_unit_final}) Put 99 if don't know

I2.b.2. Used for self-consumption by your household?

(in ${harvesting_unit_final}) Put 99 if don't know

I2.c.3. Sold?

(in ${harvesting_unit_final}) Put 99 if don't know

I2.d.4. Other uses.

(in ${harvesting_unit_final}) Put 99 if don't know

I3. How much total revenue did you earn by selling ${prim_cropname} in ${seasonnumber} ?

(in Rupees) Put 99 if don't know

${second_cropname}= value as in D2.

We are now going to ask you questions on crop ${second_cropname} that you cultivated in ${seasonnumber}:

J1. How much area did you cultivate in ${seasonnumber} under ${second_cropname} ?

(in ${land_unit_final}; including cultivation on owned land and leased land)

J2. Have you collected the harvest for ${second_cropname}?

◯ (1) Yes ◯ (2) No

J2.1. How much was the total harvest of ${second_cropname} in ${seasonnumber}?

I1.1. What was your unit of measurement for harvest of ${second_cropname} ?

◯ Tonnes ◯ Quintals ◯ Kilograms ◯ Litres ◯ Grams ◯ Bags ◯ Other

I1.2. Specify other unit.

${harvesting2_unit_final} = value as in I1.1

I1.3. How many kilograms are there in 1 ${harvesting2_unit_final}

J3. Which of the following fertilizers did you apply for cultivating ${second_cropname} in ${seasonnumber}?

(select all that apply)

☐ Urea

☐ DAP (Di-ammonium Phosphate)

☐ MOP (Muriate of Potash)

☐ SSP (Single Super Phosphate)

☐ Complex

☐ None of the above

J3.1. What was the major source of obtaining a fertilizer?

◯ (1) Primary Agricultural Cooperative Society (PACS)

◯ (2) Mandal Office

◯ (3) Private Fertilizer Shops

◯ (4) Friend/relative (Borrowed)

◯ (5) Previous year's stock (Own Fertilizer)

◯ (95) Other source

J3.2. Specify other source of obtaining a fertilizer.

Repeat the question J4 for all fertilizers selected in J3. ${fertname_sec}= value as in J4.

J4. How much ${fertname_sec} did you apply to cultivate ${second_cropname} in ${seasonnumber}?

(in kilograms) Put 99 if Don't know

J5. Which of the following inputs did you apply for cultivating ${second_cropname} in ${seasonnumber}?

(select all that apply)

☐ Zinc ☐ Boron ☐ Gypsum ☐ None of the above

J5.1. What was the major source of obtaining these inputs (i.e., Zinc, Boron, Gypsum)?

◯ (1) Primary Agricultural Cooperative Society (PACS)

◯ (2) Mandal Office

◯ Private Fertilizer Shops

◯ (4) Borrowed from friend/relative

◯ (5) Previous year's stock (Own Zinc/Boron/Gypsum)

◯ (95) Other source

J5.1. Specify other source of obtaining these inputs.

Repeat the question J6 for all micronutrients selected selected in J5. ${microname_sec2} = value as in J5.

J6. How much ${microname_sec2} did you apply to cultivate ${second_cropname} in ${seasonnumber}?

(in kilograms) Put 99 if Don't know

J7. Did you irrigate your land to cultivate ${second_cropname} in ${seasonnumber} ?

◯ (1) Yes ◯ (2) No

Section K: Travel details

K1. How do you mostly travel to Primary Agricultural Cooperative Society (PACS)?

◯ by foot ◯ by cycle ◯ by scooter/motorcycle ◯ by car ◯ by bus ◯ by autorickshaw

◯ by tonga/bullock cart ◯ other

K1.1. Specify other mode of travel.

K2. How far is the PACS from your home?

(in kilometres) Put 99 if Don’t know

${pacsmodename}= value as in K1.

K3. How much average time does it take to reach PACS ${pacsmodename}?

(in minutes)

K4. How much is the average total cost of travelling to PACS ${pacsmodename} (including cost of transporting inputs from PACS to home)? (enter the cost of 1 full trip of going to PACS and then coming back home)

(in Rupees) Put 99 if don’t know

K5. How often is Zinc/Boron/Gypsum available at PACS?

◯ (1) Never ◯ (2) Rarely ◯ (3) Sometimes ◯ (4) Mostly ◯ (5) Always

K6. How much time do you spend waiting in queue to buy Zinc/Boron/Gypsum from PACS?

(in minutes)

K7. How often is a fertilizer (Urea, DAP, MOP etc.) available at PACS?

◯ (1) Never ◯ (2) Rarely ◯ (3) Sometimes ◯ (4) Mostly ◯ (5) Always

K8. How do you mostly travel to Mandal Agriculture Office?

◯ by foot ◯ by cycle ◯ by scooter/motorcycle ◯ by car ◯ by bus ◯ by autorickshaw

◯ by tonga/bullock cart ◯ other

K8.1. Specify other mode of travel.

K9. How far is the Mandal office from your home?

(in kilometres) Put 99 if Don’t know

${officemodename}=value as in K8.

K10. How much average time does it take to reach Mandal Office by ${officemodename} ?

(in minutes)

K11. How much is the average total cost of travelling to Mandal office ${officemodename} (including cost of transporting inputs from Mandal Office to home) ? (enter the cost of 1 full trip of going to Mandal Office and then coming back home)

(in Rupees) Put 99 if don't know

K12. How often is Zinc/Boron/Gypsum available at Mandal Office?

◯ (1) Never ◯ (2) Rarely ◯ (3) Sometimes ◯ (4) Mostly ◯ (5) Always

K13. How much time do you spend waiting in queue to buy Zinc/Boron/Gypsum from Mandal Office?

(in minutes)

K14. How often is a fertilizer (Urea, DAP, MOP etc.) available at Mandal Office?

◯ (1) Never ◯ (2) Rarely ◯ (3) Sometimes ◯ (4) Mostly ◯ (5) Always

K15. How do you mostly travel to Fertilizer Shop?

◯ by foot ◯ by cycle ◯ by scooter/motorcycle ◯ by car ◯ by bus ◯ by autorickshaw

◯ by tonga/bullock cart ◯ other

K15.1. Specify other mode of travel.

K16. How far is the Fertilizer Shop from your home?

(in kilometres) Put 99 if Don't know

${shopmodename} =value as in K15.

K17. How much average time does it take to reach Fertilizer Shop by ${shopmodename}?

(in minutes)

K18. How much is the average total cost of travelling to Fertilizer Shop ${shopmodename} (including cost of transporting inputs from Fertilizer Shops to home) ? (enter the cost of 1 full trip of going to Mandal Office and then coming back home)

(in Rupees) Put 99 if don't know

K19. How often is Zinc/Boron/Gypsum available at Fertilizer Shop?

◯ (1) Never ◯ (2) Rarely ◯ (3) Sometimes ◯ (4) Mostly ◯ (5) Always

K20. How much time do you spend waiting in queue to buy Zinc/Boron/Gypsum from Fertilizer Shop?

(in minutes)

K21. How often is a fertilizer (Urea, DAP, MOP etc.) available at Fertilizer Shop?

◯ (1) Never ◯ (2) Rarely ◯ (3) Sometimes ◯ (4) Mostly ◯ (5) Always

M1. Did you purchase any input on credit?

◯ (1) Yes ◯ (2) No

M2. (if M1=1) Which of the following inputs did you buy on credit?

(select all that apply)

☐ (1) Zinc ☐ (2) Boron ☐ (3) Gypsum ☐ (4) Urea ☐ (5) DAP ☐ (6) MOP ☐ (7) Complex ☐ (8) TSP

☐ (9) SSP ☐ (10) Seeds ☐ (11) Pesticides ☐ (12) Weedicides ☐ (13) Herbicides ☐ (14) Insecticides

☐ (15) Machinery ☐ (95) Others

M2.1. Specify other inputs purchased on credit.

We are now going to ask you questions about the asset ownership of your household.

N1. Which of the following assets does your household own?

(select all that apply)

Dairy cow	◯ (1) Yes ◯ (2) No
Bull/bullock	◯ (1) Yes ◯ (2) No
Buffalo	◯ (1) Yes ◯ (2) No
Goat	◯ (1) Yes ◯ (2) No
Sheep	◯ (1) Yes ◯ (2) No
Chicken	◯ (1) Yes ◯ (2) No
Duck	◯ (1) Yes ◯ (2) No
Shallow/borewell (in field)	◯ (1) Yes ◯ (2) No

Tubewell (at home) ◯ (1) Yes ◯ (2) No

Power tiller (2 wheel-tractor) ◯ (1) Yes ◯ (2) No

Large tractor (4 wheels) ◯ (1) Yes ◯ (2) No

Thresher (electric or manual) ◯ (1) Yes ◯ (2) No

Combined harvester/thresher ◯ (1) Yes ◯ (2) No

Sprayer ◯ (1) Yes ◯ (2) No

Rice miller ◯ (1) Yes ◯ (2) No

Refrigerator ◯ (1) Yes ◯ (2) No

Radio ◯ (1) Yes ◯ (2) No

Television ◯ (1) Yes ◯ (2) No

Electric fan ◯ (1) Yes ◯ (2) No

Bed ◯ (1) Yes ◯ (2) No

Chair ◯ (1) Yes ◯ (2) No

Mobile phone ◯ (1) Yes ◯ (2) No

Pedal/cycle rickshaw ◯ (1) Yes ◯ (2) No

Autorickshaw ◯ (1) Yes ◯ (2) No

Motorcycle ◯ (1) Yes ◯ (2) No

Car ◯ (1) Yes ◯ (2) No

Van ◯ (1) Yes ◯ (2) No

Fish pond ◯ (1) Yes ◯ (2) No

O2. Please rank the following sources of information in descending order of trust you place to obtain information and advice on fertilizer, micronutrients, seeds and other agricultural inputs or issues:-

O2a. 1st rank (Most trustworthy source of information used by you)

(Select one)

◯ (1) Multi-Purpose Extension Officers (MPEOs)

◯ (2) Extension Personnel of Department of Agriculture

◯ (3) Fertilizer Retailers/dealers (shops)

◯ (4) Newspaper

◯ (5) Radio

◯ (6) Mobile Phone

◯ (7) Other farmers in contact (friends and/or relatives)

◯ (8) Personal Experience

◯ (9) TV

O2b. 2nd rank (2nd most trustworthy source of information used by you)

(Select one)

◯ (1) Multi-Purpose Extension Officers (MPEOs)

◯ (2) Extension Personnel of Department of Agriculture

◯ (3) Fertilizer Retailers/dealers (shops)

◯ (4) Newspaper

◯ (5) Radio

◯ (6) Mobile Phone

◯ (7) Other farmers in contact (friends and/or relatives)

◯ (8) Personal Experience

◯ (9) TV

◯ (95) Other sources

◯ (777) None

O2.b.1. Specify other source.

O2.c. 3rd rank (3rd most trustworthy source of information used by you)

(Select one)

◯ (1) Multi-Purpose Extension Officers (MPEOs)

◯ (2) Extension Personnel of Department of Agriculture

◯ (3) Fertilizer Retailers/dealers (shops)

◯ (4) Newspaper

◯ (5) Radio

◯ (6) Mobile Phone

◯ (7) Other farmers in contact (friends and/or relatives)

◯ (8) Personal Experience

◯ (9) TV

◯ (95) Other sources

◯ (777) None

O2.b.1. Specify other source.

P1n1. Did you give any fingerprint in any of your purchase of fertilizer or micronutrient (Zinc, Boron, Gypsum) in Kharif 2018 or Rabi 2018-19?

◯ (1) Yes ◯ (2) No

If P1n1=1, then ask P1- P3.1

P1. How frequently do you face errors while giving fingerprint?

◯ (1) Mostly ◯ (2) Sometimes ◯ (3) Rarely ◯ (4) Never ◯ (93) Don't remember ◯ (-99) Don't know

P2. What is the average time taken during finger printing?

(in minutes)

◯ (1) less than 1 minute ◯ (2) 1 - 5 minutes ◯ (3) 5-10 minutes ◯ (4) more than 10 minutes

◯ (93) Don't remember ◯ (-99) Don't know

P3. What is the average number of times you have to give fingerprint for a successful transaction?

◯ (1) 1 ◯ (2) 2 ◯ (3) 3 ◯ (4) more than 3 ◯ (93) Don't remember ◯ (-99) Don't know

P3.1. Do you think giving fingerprint has made buying fertilizer/micronutrient (Zinc, Boron, Gypsum etc) easier for you?

◯ (1) Disagree ◯ (2) Somewhat agree ◯ (3) Neutral ◯ (4) Agree ◯ (5) Strongly Agree ◯ (-99) Don't know

E3.2. By whom did you get to know about Zinc first?

☐ (1) Multi-Purpose Extension Officers (MPEOs)

☐ (2) Extension Personnel of Department of Agriculture

☐ (3) Fertilizer Retailers/dealers (shops)

☐ (4) Newspaper

☐ (5) Radio

☐ (6) Mobile Phone

☐ (7) Other farmers in contact (friends and/or relatives)

☐ (8) Personal Experience

☐ (95) Other sources

E3.2.1. Specify other

P3.3. Do you remember the year in which you started using Zinc?

◯ (1) Yes ◯ (2) No

P3.4. (if P3.3=1) In which year did you start using Zinc?

Please enter year greater than 1950 and less than 2020.

P4. Are you able to identify zinc deficiency in your soil?

◯ (1) Yes ◯ (2) No

P5. (if P4=1) Which of the following point to zinc deficiency?

(select all that apply)

☐ (1) Insufficient growth of the crop

☐ (2) Fewer panicles in crop

☐ (3) Brown spots on upper leaves

☐ (95) Other

P5.1. Specify other.

P6. Why do you think you need to apply Zinc if there is a deficiency of Zinc in your crop?

(select all that apply)

☐ (1) The input dealer asked to apply

☐ (2) it will increase the yields

☐ (3) It will reduce the application of other fertilizers

☐ (4) MPEO/ AEO asked to apply

☐ (5) Friends/ relatives asked to apply

☐ (95) Others

☐ (-99) Don’t know

P6.1. Specify other.

P7. Can Zinc be applied along with other fertilizers to the crop?

◯ (1) yes ◯ (2) no ◯ (93) don’t remember ◯ (-99) don’t know

P8. How does Zinc need to be applied?

◯ (1) Mixed with water and applied as spray after crop development

◯ (2) Mixed into soil before sowing of crop

◯ (3) Mixed with seeds of crop before sowing

◯ (95) Others

◯ (-99) Don’t know

P8.1. Specify other.

P9. How often does an Extension Personnel of Department of Agriculture visit your village in a month? (in days)

◯ (1) never visits

◯ (2) everyday

◯ (3) twice a month

◯ (4) thrice a month

◯ (5) 4 times a month

◯ (6) more than 4 times a month

◯ (7) Once a month

◯ (-99) don’t know

P10. Do you know who the officer/MPEO for your village is?

◯ (1) Yes ◯ (2) No

P11. (if P10=1) Please specify the name of the MPEO who contacted you last time

Put 99 if Don’t know

P12. Do you know where is the nearest Soil testing Lab from village?

◯ (1) Yes ◯ (2) No

P13. Do you know if any soil sample was collected from your village in the last 1 year?

◯ (1) Yes ◯ (2) No

P14. Was any soil sample taken from your land in the last 1 year?

◯ (1) Yes ◯ (2) No

P15. Do you own an Aadhar Card?

◯ (1) Yes ◯ (2) No

P17. Have you heard about the Soil Health Card Scheme?

◯ (1) Yes ◯ (2) No

P18. Do you own a Soil Health Card?

(Please show the picture of soil health card to the farmer)

◯ (1) Yes ◯ (2) Yes, verified ◯ (0) No

P18.1. Do you remember the year in which you received your Soil health card?

◯ (1) Yes ◯ (2) No

P19. (if P18.1=1) In which year did you receive your Soil Health Card?

P20. Do you think it is useful to you?

◯ (1) Yes ◯ (2) No ◯ (-99) Don't know

P22. Do you understand the information provided by the Soil Health Card related to various agricultural matters?

◯ (1) Yes ◯ (2) No

P23. What are the key contents of the soil health card?

☐ (1) Status of NPK in soil

☐ (2) Status of Zinc, Boron, Gypsum in the soil

☐ (3) Status of Manganese, Iron, Copper etc. in the soil

☐ (4) Status of Physical characteristics of the soil like Acid content

☐ (5) Information about health of the soil

☐ (6) Information on how much nutrients to apply

☐ (95) Other

P23.1. Specify Other 1

R. I am now going to list out some external events. Please let me know if any of these have had a SIGNIFICANT impact on your income in the last two years (after 2016)

R1.1. Flood ◯ (1) Yes ◯ (2) No

R1.2. Excessive rain ◯ (1) Yes ◯ (2) No

R1.3. Inadequate rainfall/drought ◯ (1) Yes ◯ (2) No

R1.4. Storm/cyclone ◯ (1) Yes ◯ (2) No

R1.5. Disease/insect/animal related shock ◯ (1) Yes ◯ (2) No

R1.6. Fire ◯ (1) Yes ◯ (2) No

R1.7. Demonetization ◯ (1) Yes ◯ (2) No

S . I am now going to ask you a few questions about your perceptions of certain events around you. There are no right or wrong answers to these questions and we only want to know how you feel about certain events. For example, you may think that you have no control over sending your child to a school , but you may have some control over what will be cooked for dinner in the house tonight. I will ask you to rank your responses from (1)lowest to (6)highest. If you think you have complete control over sending your child to school, then you can select the highest value '6'. If you think you have some control over this event, you can select '3' or '4' and if you think you have no control over this, then select '1'. Now I will ask you another example question and please let me know your answer.

S0. To what extent do you think you can control the selling price of Paddy in the market?

◯ 1 ◯ 2 ◯ 3 ◯ 4 ◯ 5 ◯ 6

Sn1. Now, please rate the following questions in a similar manner.

S1. My life is determined by my own actions

◯ 1 ◯ 2 ◯ 3 ◯ 4 ◯ 5 ◯ 6

S2. When I get what I want, it is usually because I worked hard for it.

◯ 1 ◯ 2 ◯ 3 ◯ 4 ◯ 5 ◯ 6

S3. I am usually able to protect my personal interests.

◯ 1 ◯ 2 ◯ 3 ◯ 4 ◯ 5 ◯ 6

S4. I can mostly determine what will happen in my life.

◯ 1 ◯ 2 ◯ 3 ◯ 4 ◯ 5 ◯ 6

S5. When I make plans. I am almost certain/guaranteed/sure to make them work.

◯ 1 ◯ 2 ◯ 3 ◯ 4 ◯ 5 ◯ 6

Sn2. Now, please rate the following questions in a similar manner.

S6. To a great extent my life is controlled by accidental/chance happenings.

◯ 1 ◯ 2 ◯ 3 ◯ 4 ◯ 5 ◯ 6

S7. I feel like what happens in my life is determined by others.

◯ 1 ◯ 2 ◯ 3 ◯ 4 ◯ 5 ◯ 6

S8. It is not always wise for me to plan too far ahead because many things turn out to be a matter of good or bad fortune.

◯ 1 ◯ 2 ◯ 3 ◯ 4 ◯ 5 ◯ 6

S9. My life is chiefly controlled by other powerful people.

◯ 1 ◯ 2 ◯ 3 ◯ 4 ◯ 5 ◯ 6

S10. People like myself have little chance of protecting personal interest.

◯ 1 ◯ 2 ◯ 3 ◯ 4 ◯ 5 ◯ 6

R. I am now going to ask you a few questions about your perceptions of certain events around you. There are no right or wrong answers to these questions and we only want to know how you feel about certain events. For example, you may think that you have no control over sending your child to a school but you may have some control over what will be cooked for dinner in the house tonight. I will ask you to rank your responses from (1) lowest to (6) highest. If you think you have complete control over sending your child to school, then you can select the highest value '6'. If you think you have some control over this event, you can select '3' or '4' and if you think you have no control over this, then select '1'. Now I will ask you another example question and please let me know your answer.

R0. To what extent do you think you can control the selling price of Paddy in the market?

◯ 1 ◯ 2 ◯ 3 ◯ 4 ◯ 5 ◯ 6

Rn1. Now, please rate the following questions in a similar manner.

R1. My life is determined by my own actions

◯ 1 ◯ 2 ◯ 3 ◯ 4 ◯ 5 ◯ 6

R2. When I get what I want, it is usually because I worked hard for it.

◯ 1 ◯ 2 ◯ 3 ◯ 4 ◯ 5 ◯ 6

R3. I am usually able to protect my personal interests.

◯ 1 ◯ 2 ◯ 3 ◯ 4 ◯ 5 ◯ 6

R4. I can mostly determine what will happen in my life.

◯ 1 ◯ 2 ◯ 3 ◯ 4 ◯ 5 ◯ 6

R5. When I make plans, I am almost certain/guaranteed/sure to make them work.

◯ 1 ◯ 2 ◯ 3 ◯ 4 ◯ 5 ◯ 6

Rn2. Now, please rate the following questions in a similar manner.

R6. To a great extent my life is controlled by accidental/chance happenings.

◯ 1 ◯ 2 ◯ 3 ◯ 4 ◯ 5 ◯ 6

R7. I feel like what happens in my life is determined by others.

◯ 1 ◯ 2 ◯ 3 ◯ 4 ◯ 5 ◯ 6

R8. It is not always wise for me to plan too far ahead because many things turn out to be a matter of good or bad fortune.

◯ 1 ◯ 2 ◯ 3 ◯ 4 ◯ 5 ◯ 6

R9. My life is chiefly controlled by other powerful people.

◯ 1 ◯ 2 ◯ 3 ◯ 4 ◯ 5 ◯ 6

R10. People like myself have little chance of protecting personal interest.

◯ 1 ◯ 2 ◯ 3 ◯ 4 ◯ 5 ◯ 6

S. I am now going to list out some external events. Please let me know if any of these have had a SIGNIFICANT impact on your income in the last two years (after 2016)

S1.1. Flood	◯ (1) Yes ◯ (2) No
S1.2. Excessive rain	◯ (1) Yes ◯ (2) No
S1.3. Inadequate rainfall/drought	◯ (1) Yes ◯ (2) No
S1.4. Storm/cyclone	◯ (1) Yes ◯ (2) No
S1.5. Disease/insect/animal related shock	◯ (1) Yes ◯ (2) No
S1.6. Fire	◯ (1) Yes ◯ (2) No
S1.7. Demonetization	◯ (1) Yes ◯ (2) No

T1. Please collect the GPS coordinates

Optional, but please wait for 15-20 seconds to capture location on the phone

A12. Interviewers: Team ID

T2. Interviewers: Write any notes that data analysis or researchers should know.
