# Supplementary material for: Designing better input support programs: Lessons from zinc subsidies in Andhra Pradesh, India
Source: PLoS One. 2020 Dec 3;15(12):e0242161. doi: 10.1371/journal.pone.0242161 (PMC7714421; doi:10.1371/journal.pone.0242161)
Supplement: S2 File — (PDF) [file pone.0242161.s004.pdf]

Input Dealer survey

A1. Enumerator ID

◯ 1

◯ 2

◯ 3

◯ 4

◯ 5

◯ 6

◯ 7

◯ 8

◯ 9

◯ 10

Information and informed consent

I am …………… from Neerman – an agency doing research in the state of Andhra Pradesh. You are being asked to participate in this survey being conducted by the International Food Policy Research Institute (IFPRI- a research institute engaged in agricultural research). We are doing this survey to find out about your role in the distribution of fertilizers and micronutrients in your area.

You have been selected randomly for this survey, which is being conducted across 6 selected districts of Andhra Pradesh (deleted)among fertilizer dealers. Your participation in this survey will take about 20 minutes. If you have any questions about the survey, feel free to ask them at any point.

Before we start we would like you to know that:

• You can participate if you want to. You have the right to withdraw from this survey at any point during the survey without giving reason. If you do not wish to participate at all, you have the alternative of quitting now.

• By participating in this survey, you will not face any problems. We expect that the information obtained from this survey will be helpful in reforming the micronutrient schemes (might be useful to the Government of Andhra Pradesh and the Government of India).

• Whether you participate in this survey or not, you will neither gain nor lose anything.

• You have the right to confidentiality regarding the privacy of your personal information.

• Whatever information that you are going to tell us, we won’t tell anyone else. Only survey team will know about the responses you provide.

• We are getting this information from several participants like you, and we are going to prepare a report based on all this information, and no one (deleted) farmer’s name/village details will be shared.

Compensation:

We are not going to provide you any compensation for participating in this survey.

In case of any questions/conflicts, please contact Mr Amit Patil at 022-24021757, who will put you in touch with the research team.

A2. Do you consent to participate in the survey?

◯ (1) Yes

◯ (0) No

A4. District where the shop is located ◯ district

A5. Mandal where the shop is located ◯ mandal

A6. Village where the shop is located ◯ village

A7. Please select the place where the survey is being conducted

◯ (1) At the shop (that is in the shop premises including inside the shop, or just outside it)

◯ (95) Other place

A7(TEXT). Specify the other place where this survey is being conducted

B6. Respondent number

(Please note that the unique ID for this respondent is ${respondent_id})

B2(a). What is the name of your shop?

B2. Name of the respondent

B3. Father's name of the respondent

B4. Gender of the respondent

◯ (1) Female

◯ (2) Male

◯ (95) Other

B5. Age of the respondent

(in years)

B6. Contact number

(please enter your 10-digit mobile number)

B7. Alternative contact number

(please enter your 10-digit mobile number or your landline number)

Y1. Caste of the respondent

◯ (1) General Caste

◯ (2) Scheduled Caste

◯ (3) Scheduled Tribe

◯ (4) Other Backward Class

◯ (95) Other

Y1(TEXT-95). Specify other caste

Y2. Religion of the respondent

◯ (1) Hindu

◯ (2) Muslim

◯ (3) Sikh

◯ (4) Christian

◯ (5) Buddhist

◯ (6) Jain

◯ (95) Other

Y2(TEXT). Specify other religion

Y3. Marital status of the respondent

◯ (1) Single/Unmarried (never married)

◯ (2) Married

◯ (3) Engaged

◯ (4) Widow or widower

◯ (5) Divorced

◯ (6) Separated (w/o divorce) or deserted

Y4. What is the highest level of schooling acquired by the respondent?

◯ (1) No schooling

◯ (2) Primary (class 1-5) Completed

◯ (3) ME (class 6-7) Completed

◯ (4) High school (class 8-10) Completed

◯ (5) CHSE (class 11-12) Completed

◯ (6) Undergraduate (Bachelors) Completed

◯ (7) Postgraduate (Masters) or higher Completed

◯ (8) Vocational training/ Diploma

Y4.1. What is the literacy level of the respondent?

◯ (1) Cannot read or write

◯ (2) Can write but cannot read

◯ (3) Can read but cannot write

◯ (4) Can read and write

Y5. What was your major subject of specialization?

(enter text)

Y6. What type of a retailer are you?

◯ (1) Private dealer

◯ (2) Cooperative

Y7. Which of the following agricultural inputs do you deal in?

(select all that apply)

☐ (1) Seeds

☐ (2) Fertilizers (Urea,DAP,MOP etc.)

☐ (3) Pesticides

☐ (4) Micronutrients (Zinc, Boron, Gypsum, etc.)

☐ (5) Farm tools and implements

☐ (6) Farm machinery (mini-tractor, rice transplanter, laser land leveller, etc.)

☐ (7) Herbicides

☐ (8) Weedicides

☐ (95) Other inputs

Y7(TEXT). Specify other input

Y8. Do you distribute subsidized/free micronutrients at your store?

◯ (1) Yes

◯ (0) No

Y9. Do you sell unsubsidized micronutrients at your store?

◯ (1) Yes

◯ (0) No

Y10. Please allocate your average total sales (business volume) into the following categories:

(in percentage)

Y10(a). Seeds (in percentage)

Y10(b). Fertilizers (in percentage)

Y10(c). Micronutrients (in percentage)

Y10(d). Pesticides, weedicides and herbicides (in percentage)

Y10(e). Others (in percentage)

Y11. Which of the following are your major day-to-day-activities?

(select all that apply)

☐ (1) Hold stocks of seeds, fertilizers and micronutrients and other inputs

☐ (2) Maintain records of the total sales during the day

☐ (3) Identify the shortage of fertilizers and other inputs

☐ (4) Keep track of competing brands of fertilizers, pesticides, micronutrients and other inputs.

☐ (5) Provide inputs on credit to farmers

☐ (6) Provide money loans to farmers

☐ (7) Provide recommendations to the farmers about the right dose of inputs to be applied

☐ (8) Provide information to farmers with regard to subsidy schemes/incentives/farm equipment provided by Department of Agriculture under different programmes and educate the farmers to make use of these facilities.

☐ (9) Educating the farmers about correct agricultural practices

☐ (10) Collect soil samples for soil testing

☐ (11) Maintain scheme implementation records in your area

☐ (12) Keep track of farmers' complaints about product quality.

☐ (95) Others

Y11(TEXT). Specify other task

Y12. When did you start working here?

Month: Year:

Y13. What is the average number of hours you work in a day?

(in hours)

◯ (1) 0-4 hours

◯ (2) 4-6 hours

◯ (3) 6-8 hours

◯ (4) 8-10 hours

◯ (5) 10-12 hours

Y14. Which of the following brands of fertilizers and micronutrients do you keep mostly?

(select all that apply)

Cormandal International Ltd.	◯ (1) Yes ◯ (0) No
Indian Potash Ltd.	◯ (1) Yes ◯ (0) No
IFFCO	◯ (1) Yes ◯ (0) No
Rastriya Chemicals & Fertilizers	◯ (1) Yes ◯ (0) No
KRIBCHO	◯ (1) Yes ◯ (0) No

Fertilizers & Chemicals of Travencore ◯ (1) Yes ◯ (0) No

Gujarath Nramadha Fertilizers & Chemicals (GNFC) ◯ (1) Yes ◯ (0) No

Gujarath State Fertilizers Chemicals (GSFC) ◯ (1) Yes ◯ (0) No

Paradeep Phosphate Ltd. (PPL) ◯ (1) Yes ◯ (0) No

Manglore Chemicals & Fertilizers (MCF) ◯ (1) Yes ◯ (0) No

Nagarjuna Fertilizers & Chemical Ltd. (NFCL) ◯ (1) Yes ◯ (0) No

Green Star Fertilizers Ltd. (SPIC) ◯ (1) Yes ◯ (0) No

Zuari Agro Chemicals Ltd ◯ (1) Yes ◯ (0) No

Subhodaya Chemicals Ltd ◯ (1) Yes ◯ (0) No

National Fertilizer Ltd (NFL) ◯ (1) Yes ◯ (0) No

Madras Fertilizer Ltd ◯ (1) Yes ◯ (0) No

Aries Chalamine Gold ◯ (1) Yes ◯ (0) No

Other company ◯ (1) Yes ◯ (0) No

Y14(TEXT). Specify other brand

Y15. Which of the following brands of Zinc are most popular among the farmers in your area (select even those brands that you don’t keep at your shop but the brand is popular among the farmers)?

(select all that apply)

☐ (1) Cormandal International Ltd.

☐ (2) Indian Potash Ltd.

☐ (3) IFFCO

☐ (4) Rastriya Chemicals & Fertilizers

☐ (5) KRIBCHO

☐ (6) Fertilizers & Chemicals of Travencore

☐ (7) Gujarath Nramadha Fertilizers & Chemicals (GNFC)

☐ (8) Gujarath State Fertilizers Chemicals (GSFC)

☐ (9) Paradeep Phosphate Ltd. (PPL)

☐ (10) Manglore Chemicals & Fertilizers (MCF)

☐ (11) Nagarjuna Fertilizers & Chemical Ltd. (NFCL)

☐ (12) Green Star Fertilizers Ltd. (SPIC)

☐ (13) Zuari Agro Chemicals Ltd

☐ (14) Subhodaya Chemicals Ltd

☐ (15) National Fertilizer Ltd (NFL)

☐ (16) Madras Fertilizer Ltd

☐ (17) Aries Chalamine Gold

☐ (95) Other company

Y15(TEXT). Specify other brand

Y16. Which of the following brands of Boron are most popular among the farmers in your area (select even those brands that you don’t keep at your shop but the brand is popular among the farmers)?

(select all that apply)

☐ (1) Cormandal International Ltd.

☐ (2) Indian Potash Ltd.

☐ (3) IFFCO

☐ (4) Rastriya Chemicals & Fertilizers

☐ (5) KRIBCHO

☐ (6) Fertilizers & Chemicals of Travencore

☐ (7) Gujarath Nramadha Fertilizers & Chemicals(GNFC)

☐ (8) Gujarath State Fertilizers Chemicals(GSFC)

☐ (9) Paradeep Phosphate Ltd.(PPL)

☐ (10) Manglore Chemicals & Fertilizers(MCF)

☐ (11) Nagarjuna Fertilizers & Chemical Ltd.(NFCL)

☐ (12) Green Star Fertilizers Ltd.(SPIC)

☐ (13) Zuari Agro Chemicals Ltd

☐ (14) Subhodaya Chemicals Ltd

☐ (15) National Fertilizer Ltd (NFL)

☐ (16) Madras Fertilizer Ltd

☐ (17) Aries Chalamine Gold

☐ (95) Other company

Y16(TEXT). Specify other brand

Y17. Did you receive any training from any manufacturer to facilitate sales of any of the Zinc brands that you keep?

◯ (1) Yes

◯ (0) No

Y18. Which of the following sources do you mostly use to determine whether to increase or decrease the stocks of a certain input?

(select all that apply)

☐ (1) By asking farmers who visit here

☐ (2) Based on information collected from Agricultural Extension Services

☐ (3) By visiting the villages in your area yourself

☐ (4) By orders directed from the government

☐ (5) By orders of private companies

☐ (95) Other sources

Y18(TEXT). Specify other source

Y19. Do you use POS machine for processing transactions? (For fingerprinting)

◯ (1) Yes

◯ (0) No

Y20. (if Y19=0) Why don't you use a POS machine for processing transactions?

(select all that apply)

☐ (1) There are server issues

☐ (2) There are defects in the machine itself

☐ (3) Most farmers don’t have Aadhar card

☐ (4) It reduces the number of sales made during a day due to increased transaction time

☐ (95) Other reason

Y20(TEXT). Specify other reason

Y21. (if Y19=1) Since when had you been using the POS machine?

Month: Year:

Y22. (if Y19=1) What issues do you face while using the POS machine?

(select all that apply)

☐ (1) Server and connectivity problems

☐ (2) Multiple errors in Aadhar authentication like finger printing, etc

☐ (3) Machine works slow

☐ (4) Unavailability of Aadhar card with the farmers at the time of transaction

☐ (5) Unavailability of Soil Health Card with the farmers at the time of transaction

☐ (6) Lack of time to handle all the transactions due to a lot of farmers

☐ (7) Lack of time to handle all the transactions due to other works at the store

☐ (95) Others

Y22(TEXT). Specify other issues

Y23. (if Y19=1) How would you rate the network connectivity in POS machines?

◯ (1) Poor

◯ (2) Weak

◯ (3) Average

◯ (4) Strong

◯ (5) Excellent

◯ (-91) Cant say

Y24. (if Y19=1) What do you do when the machine doesn't work?

(select all that apply)

☐ (1) Record the name and details of the farmer before making sales in a register only

☐ (2) Record the name and details of the farmer before making sales in a register and enter it in machine later

☐ (3) Make the sale without keeping any record

☐ (4) Wait until the machine starts working again and only then make the sales

☐ (95) Other

Y24(TEXT). Specify other

Y25. Which mechanism do you use to report operational and technical issues for the POS machine? (select all that apply)

☐ (1) Report to the related person using Whatsapp

☐ (2) Report online at the government portal

☐ (3) Do not report and fix yourself

☐ (4) Do not report and wait for the issue to get fixed on it own

☐ (95) Other

Y25(TEXT). Specify other mechanism.

Y26. How often do you encounter biometric authentication errors at the time of purchase?

◯ (1) Mostly

◯ (2) Sometimes

◯ (3) Rarely

◯ (4) Never

Y27. What is the average time taken during an Aadhar enabled transaction?

◯ (1) less than 1 minute

◯ (2) 1 - 5 minutes

◯ (3) 5-10 minutes

◯ (4) more than 10 minutes

◯ (5) Don’t remember

Y28. What is the average number of attempts needed for successful biometric authentication for a transaction?

◯ (1) 1

◯ (2) 2

◯ (3) 3

◯ (4) more than 3

◯ (5) Don't remember

Y29. Do you think using POS machines has facilitated you in increasing sales of agricultural inputs to farmers?

◯ (1) Disagree

◯ (2) Somewhat disagree

◯ (3) Neutral

◯ (4) Agree

◯ (5) Strongly Agree

Y30. During the start of any season (peak season), how many farmers do you cater to on an average per day?

Y31. What proportion of farmers out of the ${number_farmer} that you cater to provide Aadhar card at the time of purchase?

(in percentage)

Y32. What proportion of farmers out of the ${number_farmer} that you cater to bring or possess a Soil Health Card?

(in percentage)

Y33. What proportion of farmers out of the ${number_farmer} that you cater to take micronutrients at subsidy?

(in percentage)

Y34. What proportion of farmers out of the ${number_farmer} that you cater to buy micronutrients (like zinc, boron, gypsum,etc) not on subsidy?

(in percentage)

Y35. What proportion of farmers out of the ${number_farmer} that you cater to obtain Free Zinc from you?

(in percentage)

Y36. Do you face any shortage of any input (like seeds, fertilizers, micronutrients, etc)?

◯ (1) Yes

◯ (0) No

Y37. (if Y36=1) For which of the following inputs do you face a shortage?

(select all that apply)

☐ (1) Seeds

☐ (2) Urea

☐ (3) DAP

☐ (4) MOP

☐ (95) Other fertilizers

☐ (6) Zinc

☐ (7) Boron

☐ (8) Gypsum

☐ (96) Other micronutrient

☐ (10) Pesticides

☐ (11) Farm tools and implements

☐ (12) Farm machinery (mini-tractor, rice transplanter, laser land leveller, etc.)

☐ (13) Herbicides

☐ (14) Weedicides

☐ (97) Other inputs

Y38. How often do you get complaints from the farmers regarding the quality of fertilizers?

◯ (1) Mostly

◯ (2) Sometimes

◯ (3) Rarely

◯ (4) Never

Y39. How often do you get complaints from the farmers regarding the quality of micronutrients?

◯ (1) Mostly

◯ (2) Sometimes

◯ (3) Rarely

◯ (4) Never

Y40. How often do you get complaints from the farmers regarding the quality of other inputs like pesticides, farm equipment, etc ?

◯ (1) Mostly

◯ (2) Sometimes

◯ (3) Rarely

◯ (4) Never

1.Please collect the GPS coordinates (Please wait for 15-20 seconds to capture location on the phone)

2. Interviewers: Team ID

3. Interviewers: Write any notes that data analysis or researchers should know.

4. Enumerator: Please take photo of zinc bag with brand name

5. Enumerator: Please take photo of zinc bag with MRP and quantity

6. Enumerator: Please take photo of zinc bag with brand name

7. Enumerator: Please take photo of zinc bag with MRP and quantity

8. Enumerator: Please take photo of zinc bag with brand name

9. Enumerator: Please take photo of zinc bag with MRP and quantity
