# Supplementary material for: Designing better input support programs: Lessons from zinc subsidies in Andhra Pradesh, India
Source: PLoS One. 2020 Dec 3;15(12):e0242161. doi: 10.1371/journal.pone.0242161 (PMC7714421; doi:10.1371/journal.pone.0242161)
Supplement: S3 File — (PDF) [file pone.0242161.s005.pdf]

Extension worker survey

Q1. Enumerator ID

◯ 1

◯ 2

◯ 3

◯ 4

◯ 5

◯ 6

◯ 7

◯ 8

◯ 9

◯ 10

Consent form:

Greetings! I am …………… from Neerman – an agency doing research in the state of Andhra Pradesh. You are being asked to participate in this survey being conducted by the International Food Policy Research Institute (IFPRI- a research institute engaged in agricultural research). We are doing this survey to find out about your activities and responsibilities in the Mandal Agricultural office. In this survey we will ask you questions on your daily tasks and about the micronutrient subsidy scheme of the government of Andhra Pradesh.

You have been selected randomly for this survey, which is being conducted across 6 selected districts of Andhra Pradesh among MPEO's and AEO's. Your participation in this survey will take about 20 minutes. If you have any questions about the survey, feel free to ask them at any point.

Before we start we would like you to know that: • You can participate if you want to. You have the right to withdraw from this survey at any point during the survey without giving reason. If you do not wish to participate at all, you have the alternative of quitting now. • By participating in this survey, you will not face any problems. We expect that the information obtained from this survey will be helpful in reforming the micronutrient schemes (might be useful to the Government of Andhra Pradesh and the Government of India). • Whether you participate in this survey or not, you will neither gain nor lose anything. • You have the right to confidentiality regarding the privacy of your personal information. • Whatever information that you are going to tell us, we won't tell anyone else. Only survey team will know about the responses you provide. • We are getting this information from several participants like you, and we are going to prepare a report based on all this information, and no one name/village details will be shared.

Compensation: We are not going to provide you any compensation for participating in this survey. In case of any questions/conflicts, please contact Mr Amit Patil at 022-24021757, who will put you in touch with the research team.

Q2. Do you consent to participate in the survey?

◯ (1) Yes

◯ (0) No

A1. District where the survey is conducted ◯ district

A2. Mandal where the survey is conducted ◯ mandal

A3. Village where the survey is conducted ◯ village

A4. Please select the place where this survey is being conducted

◯ (1) At PACS

◯ (2) At Mandal Office

◯ (95) Other Place

A4(TEXT). Specify other place

A5. Respondent Number.

Please note that the Respondent ID for this respondent is ${respondent_id}

A7. Name of the respondent

A9. Gender of the respondent

◯ (1) Female

◯ (2) Male

◯ (95) Other

A10. Age of the respondent

(in years)

A11. Contact number

(please enter your 10-digit mobile number)

A12. Alternative contact number

(please enter your 10-digit mobile number or your landline number)

A13. Do you belong to the same Mandal in which you are posted?

◯ (1) Yes

◯ (0) No

A14. Respondent's caste

◯ (0) General Caste

◯ (1) Scheduled Caste

◯ (2) Scheduled Tribe

◯ (3) Other Backward Class

◯ (95) Other

A14(TEXT-95). Specify other caste

A14.1. Please specify respondent's jaati.

A14.1. Respondent's Jaati

◯ (0) Brahman

◯ (1) Kapu

◯ (2) Kamma

◯ (3) Komati

◯ (4) Kshatriya

◯ (95) Others

◯ (7) Madiga

◯ (8) Mala

◯ (96) Others

◯ (10) Balija

◯ (11) Boya/Besta

◯ (12) Chakali

◯ (13) Devanga

◯ (14) Dudekula

◯ (15) Goundla

◯ (16) Gavara

◯ (17) Golla

◯ (18) Idiga

◯ (19) Jangam

◯ (20) Kammara/

◯ (21) Vishwa

◯ (22) Brahmana

◯ (23) Kummari

◯ (24) Kurma

◯ (25) Munnurukapu

◯ (26) Mangali

◯ (27) Mutrasi

◯ (28) Padmasali

◯ (29) Telaga

◯ (30) Uppara

◯ (31) Waddera

◯ (32) Velama

◯ (33) Yadav

◯ (97) Others

A14.1(TEXT). Specify other jaati.

A15. Respondent's religion

◯ (1) Hindu

◯ (2) Muslim

◯ (3) Sikh

◯ (4) Christian

◯ (5) Buddhist

◯ (6) Jain

◯ (95) Other

A15(TEXT). Specify other religion

A16. What is the highest level of schooling acquired by the respondent?

◯ (1) No schooling

◯ (2) Primary (class 1-5) Completed

◯ (3) ME (class 6-7) Completed

◯ (4) High school (class 8-10) Completed

◯ (5) CHSE (class 11-12) Completed

◯ (6) Undergraduate (Bachelors) Completed

◯ (7) Postgraduate (Masters) or higher Completed

◯ (8) Diploma

◯ (9) Vocational Training

A17(1). What was your major subject of specialization?

◯ (1) Agriculture

◯ (2) Horticulture

◯ (3) Agriculture Polytechnic Diploma Holders (ANGRAU recognized)

◯ (4) BZC

◯ (95) Other

A17(2). Specify other subject.

Section B: Job description

B1. Please select the District in which your office is located. ◯ district

B2. Please select the Mandal in which you are posted. ◯ mandal

B3. In which of the following places is your office located?

◯ (1) Primary Agricultural Cooperative Society (PACS)

◯ (2) Mandal Agricultural Office

◯ (3) District Agricultural Office

◯ (95) Other

B3(TEXT). Specify other place

B4. How much time does it take you to reach office from your home by your usual mode of transport?

(in minutes)

B5. What is your designation?

◯ (1) Agricultural Extension Officer (AEO)

◯ (2) Multi-Purpose Extension Officer (MPEO)

B6. Which of the following are your day-to-day responsibilities?

(select all that apply)

☐ (1) Provide information to farmers with regard to all government schemes

☐ (2) Solve farmers problems in cultivation

☐ (3) Encourage farmers for Zero Budget Natural Farming

☐ (4) Collect soil samples for soil testing/inspection

☐ (5) Organizing demonstrations

☐ (6) Seed distribution through D-Krishi App

☐ (7) Maintain scheme implementation and farmer records in your area

☐ (8) Collect documentation of farmers for scheme implementation

☐ (9) Assist the farmers in obtaining farmer loans from the government

☐ (10) Maintain record on tenant farmer scheme.

☐ (11) CRK scheme implementation.

☐ (95) Others

B6(TEXT). Specify other task

B7. What is the total number of apps for schemes that you manage?

B8. How many farmers do you interact with everyday on an average?

(on phone and personally)

B9. How many villages are you responsible for?

B10. How many farmers are you responsible for?

B11. Please select the names of the villages that you are responsible for. ☐ village

B12. How many times do you visit a village (that you are responsible for) in a week?

Section C: Micronutrient

C1. Which of the following micronutrients are mostly used by the farmers in your villages?

(select all that apply)

☐ (1) Zinc

☐ (2) Boron

☐ (3) Gypsum

☐ (4) Chlorine

☐ (5) Copper

☐ (6) Iron

☐ (7) Manganese

☐ (8) Magnesium

☐ (95) Others

C1(TEXT). Specify other micronutrient

C2. Which of the following are the most common nutrient deficiencies in your villages?

(select all that apply)

☐ (1) Zinc

☐ (2) Boron

☐ (3) Chlorine

☐ (4) Copper

☐ (5) Iron

☐ (6) Manganese

☐ (7) Magnesium

☐ (8) Gypsum

☐ (9) Nitrogen

☐ (10) Phosphorus

☐ (11) Potash

☐ (95) Others

C2(TEXT). Specify other nutrient

D1. What are the sources of micronutreints used by farmers in your cluster (please select rankwise):

D1(1). Most important source:

◯ (1) Primary Agricultural Cooperative Society (PACS)

◯ (2) Mandal Agricultural Office

◯ (3) Fertilizer Dealer shops

◯ (95) Others

D1(1)(TEXT). Specify other.

D1(2). Second most important source:

◯ (1) Primary Agricultural Cooperative Society (PACS)

◯ (2) Mandal Agricultural Office

◯ (3) Fertilizer Dealer shops

◯ (777) None

◯ (951) Others

D1(2)(TEXT). Specify other.

D1(3). Third most important source:

◯ (1) Primary Agricultural Cooperative Society (PACS)

◯ (2) Mandal Agricultural Office

◯ (3) Fertilizer Dealer shops

◯ (777) None

◯ (952) Others

D1(3)(TEXT). Specify other.

D2. What was your target of amount of Zinc to be supplied in Kharif 2018, if any?

(in tonnes)

D3. How much Zinc did you supply in Kharif 2018?

(in tonnes)

D4. How do you identify zinc deficiency?

(select all that apply)

☐ (1) Manual inspection of the crop colour

☐ (2) Manual inspection of the field

☐ (3) Based on soil testing

☐ (4) By talking to the farmers

☐ (5) By Soil Health Card

☐ (95) Other ways

D4(TEXT). Specify other way

D5. What percentage of farmers in your cluster reported zinc deficiency?

(in percentage)

D6. How do you select the beneficiaries of Zinc subsidy?

(select all that apply)

☐ (1) Through Soil Health Card

☐ (2) By talking to farmers directly

☐ (3) By observing the crop

☐ (95) Other

D6(TEXT). Specify other

D7. What is the eligibility criterion for getting Zinc subsidy?

(select all that apply)

☐ (1) Zinc content in Soil Health Card is less thn 0.5 ppm

☐ (2) Farmer must have Loan eligible card (LEC) or Patta

☐ (3) Farmer must have Aadhar Card

☐ (95) Other

D7(TEXT). Specify other

D10. What were the main problems you faced during Zinc distribution?

(select all that apply)

☐ (0) No problem faced

☐ (1) Farmers don’t own Soil Health Cards

☐ (2) Farmers don’t have Aadhar Card

☐ (3) Farmers don’t have Certificate of Cultivation (COC)/LEC

☐ (4) Finger-print authentification error

☐ (5) Internet problem

☐ (6) Tablets don’t work

☐ (7) Not enough time to carry out all tasks

☐ (8) Problem in identification of land owners due to tenancy

☐ (9) Farmers don’t come to pick up their Zinc from PACS/Mandal office

☐ (10) Farmers don’t want to adopt Zinc

☐ (11) Huge labour costs of application

☐ (12) Demand of Zinc is more than supply

☐ (13) Excess supply of Zinc

☐ (95) Others

D10(TEXT). Specify other problem

D11. Which of the following strategies did you adopt to ovecome the problems you just mentioned?

(select all that apply)

☐ (1) Note transactions in the notebook if the farmer does not show Aadhar Card

☐ (2) Give self/officer biometric authentication

☐ (3) Use own mobile phone

☐ (4) Overtime

☐ (5) Deliver Zinc to farmers when they don’t come

☐ (6) Spread awareness among the farmers about Zinc benefits

☐ (7) Maintain stocks of Zinc to meet excess demand or manage excess supply

☐ (8) Report the problem to Central Command Wing

☐ (95) Other strategy 1

D11(TEXT-95). Please specify other strategy.

D12. Which brand of Zinc did you distribute in Kharif 2018?

(select all that apply)

☐ (1) Cormandal International Ltd.

☐ (2) Indian Potash Ltd.

☐ (3) Bhagya Zinc

☐ (4) IFFCO

☐ (5) Rastriya Chemicals & Fertilizers

☐ (6) KRIBCHO

☐ (7) Fertilizers & Chemicals of Travencore

☐ (8) Gujarath Nramadha Fertilizers & Chemicals(GNFC)

☐ (9) Gujarath State Fertilizers Chemicals(GSFC)

☐ (10) Paradeep Phosphate Ltd.(PPL)

☐ (11) Manglore Chemicals & Fertilizers(MCF)

☐ (12) Nagarjuna Fertilizers & Chemical Ltd.(NFCL)

☐ (13) Green Star Fertilizers Ltd.(SPIC)

☐ (99) Don’t know

☐ (95) Other company

D12(TEXT). Specify other brand name

D13. What chemical composition of Zinc did you supply?

(select all that apply)

☐ (1) Zinc sulfate 12%

☐ (2) Zinc sulfate 21%

☐ (3) Zinc sulfate 33%

☐ (99) Don’t know

☐ (95) Other

D13(TEXT). Specify other chemical composition of Zinc that you supplied.

D14a. What was the method of Zinc application that you mostly recommended in Kharif 2018?

◯ (1) Basal application

◯ (2) Foliar application

D14b. Is the method that you recommended, also the best method of application generally?

◯ (1) Yes

◯ (0) No

D15. How would you rate the quality of the Zinc brands that you distributed under the Micronutrient Subsidy scheme?

◯ (1) Excellent

◯ (2) Good

◯ (3) Moderate

◯ (4) Poor

◯ (5) Can't say

D16. How do you tell if a crop is zinc deficient?

(select all that apply)

☐ (1) decreased height of crop

☐ (2) fewer panicles in the crop

☐ (3) brown spots on upper leaves

☐ (4) yellowing of leaves between the veins

☐ (5) distorted leaves

☐ (6) don’t know

☐ (7) don’t remember

☐ (95) other indicator

D16(TEXT). Specify other.

D17. What are the main benefits of applying Zinc to the crop?

(select all that apply)

☐ (1) Positive impact on yield

☐ (2) Replenishes soil nutrients

☐ (3) Improves tolerance of plants to various environmental stress factors

☐ (4) Leads to better seed viability and seedling vigour

☐ (5) Reduces accumulation of Cadmium in the seeds

☐ (6) Improves the quality of the crop

☐ (7) Provides nutritional benefits for human health

☐ (95) Other benefit

D17(TEXT). Specify other benefit.

D18. Can zinc be applied alongwith other fertilizers?

◯ (1) Yes, with all the fertilizers

◯ (2) Yes but without phosphorous

◯ (3) No

◯ (4) Don’t know

D20. What is the best time to apply Zinc on field?

◯ (1) Soil preparation (Basal)

◯ (2) At the time of sowing seeds

◯ (3) During field maintenance like weeding, irrigating, applying fertilizers etc.

◯ (4) Harvesting

◯ (99) Don’t know

◯ (93) Don’t remember

◯ (95) Other

D20(TEXT). Specify other time

D21. What is right dosage of Zinc that must be applied?

(in kg per acre)

D22. How do you spread the information about the benefits of zinc in your villages?

(select all that apply)

☐ (1) Send voice or text message to farmers on phone

☐ (2) Press/radio/tv advertisement

☐ (3) Announcements in the village on a loudspeaker

☐ (4) Door to door visit to farmer's home

☐ (5) Informing groups of farmers at village level

☐ (6) Distribution of brochures and pamphlets

☐ (7) Informing farmers through Gram Sabha

☐ (95) Others

D22(TEXT). Specify other

We are now going to ask you questions on Boron in Kharif 2018

E1. What was your target of amount of boron to be supplied in Kharif 2018, if any?

(in tonnes)

E2. How much boron did you supply in Kharif 2018?

(in tonnes)

E3. How do you identify boron deficiency?

(select all that apply)

☐ (1) Manual inspection of the crop colour

☐ (2) Manual inspection of the field

☐ (3) Based on soil testing

☐ (4) By talking to the farmers

☐ (5) By Soil Health Card

☐ (95) Other ways

E3(TEXT). Specify other way

E4. What percentage of farmers in your cluster reported boron deficiency?

(in percentage)

E5. How do you tell if a crop is boron deficient?

(select all that apply)

☐ (1) decreased height of crop

☐ (2) fewer panicles in the crop

☐ (3) brown spots on upper leaves

☐ (4) yellowing of leaves between the veins

☐ (5) distorted leaves

☐ (6) don't know

☐ (7) don't remember

☐ (95) other indicator

E5(TEXT). Specify other.

E6. What are the main benefits of applying boron to the crop?

(select all that apply)

☐ (1) Positive impact on yield

☐ (2) Replenishes soil nutrients

☐ (3) Improves tolerance of plants to various environmental stress factors

☐ (4) Leads to better seed viability and seedling vigour

☐ (5) Reduces accumulation of Cadmium in the seeds

☐ (6) Improves the quality of the crop

☐ (7) Provides nutritional benefits for human health

☐ (95) Other benefit

E6(TEXT). Specify other benefit.

E7. Can boron be applied along with other fertilizers?

◯ (1) Yes, with all the fertilizers

◯ (2) Yes but without phosphorous

◯ (3) No

◯ (4) Don’t know

E8. What is the best time to apply boron on field?

◯ (1) Soil preparation (Basal)

◯ (2) At the time of sowing seeds

◯ (3) During field maintenance like weeding, irrigating, applying fertilizers etc.

◯ (4) Harvesting

◯ (99) Don’t know

◯ (93) Don’t remember

◯ (95) Other

E8(TEXT). Specify other time

E9. What is right dosage of boron that must be applied?

(in kg per acre)

We are now going to ask you questions on Gypsum in Kharif 2018

F1. What was your target of amount of gypsum to be supplied in Kharif 2018, if any?

(in tonnes)

F2. How much gypsum did you supply in Kharif 2018?

(in tonnes)

F3. How do you identify gypsum deficiency?

(select all that apply)

☐ (1) Manual inspection of the crop colour

☐ (2) Manual inspection of the field

☐ (3) Based on soil testing

☐ (4) By talking to the farmers

☐ (5) By Soil Health Card

☐ (95) Other ways

F3(TEXT). Specify other way

F4. What percentage of farmers in your cluster reported gypsum deficiency?

(in percentage)

F5. How do you tell if a crop is gypsum deficient?

(select all that apply)

☐ (1) decreased height of crop

☐ (2) fewer panicles in the crop

☐ (3) brown spots on upper leaves

☐ (4) yellowing of leaves between the veins

☐ (5) distorted leaves

☐ (6) don’t know

☐ (7) don’t remember

☐ (95) other indicator

F5(TEXT). Specify other.

F6. What are the main benefits of applying gypsum to the crop?

(select all that apply)

☐ (1) Positive impact on yield

☐ (2) Replenishes soil nutrients

☐ (3) Improves tolerance of plants to various environmental stress factors

☐ (4) Leads to better seed viability and seedling vigour

☐ (5) Reduces accumulation of Cadmium in the seeds

☐ (6) Improves the quality of the crop

☐ (7) Provides nutritional benefits for human health

☐ (95) Other benefit

F6(TEXT). Specify other benefit.

F7. Can gypsum be applied alongwith other fertilizers?

◯ (1) Yes, with all the fertilizers

◯ (2) Yes but without phosphorous

◯ (3) No

◯ (4) Don’t know

F8. What is the best time to apply gypsum on field?

◯ (1) Soil preparation (Basal)

◯ (2) At the time of sowing seeds

◯ (3) During field maintenance like weeding, irrigating, applying fertilizers etc.

◯ (4) Harvesting

◯ (99) Don’t know

◯ (93) Don’t remember

◯ (95) Other

F8(TEXT). Specify other time

F9. What is right dosage of gypsum that must be applied?

(in kg per acre)

Section G: Details on own land

G1. Do you or your family own any farmland?

◯ (1) Yes

◯ (0) No

G1a. In which state do you own this farmland?

(select all that apply)

☐ (1) Andhra Pradesh

☐ (2) Telangana

☐ (95) Other state

G2. Do you still practice farming on that land?

◯ (1) Yes

◯ (0) No

G3. Did you apply Zinc on your land in Kharif 2018?

◯ (1) Yes

◯ (0) No

G4. Which brand of Zinc did you apply?

(select all that apply)

☐ (1) Cormandal International Ltd.

☐ (2) Indian Potash Ltd.

☐ (3) Bhagya Zinc

☐ (4) IFFCO

☐ (5) Rastriya Chemicals & Fertilizers

☐ (6) KRIBCHO

☐ (7) Fertilizers & Chemicals of Travencore

☐ (8) Gujarath Nramadha Fertilizers & Chemicals(GNFC)

☐ (9) Gujarath State Fertilizers Chemicals(GSFC)

☐ (10) Paradeep Phosphate Ltd.(PPL)

☐ (11) Manglore Chemicals & Fertilizers(MCF)

☐ (12) Nagarjuna Fertilizers & Chemical Ltd.(NFCL)

☐ (13) Green Star Fertilizers Ltd.(SPIC)

☐ (99) Don't know

☐ (95) Other company

G4.1. Specify name of other brand.

G5. What was the crop for which you applied Zinc?

(select all that apply)

☐ (1) Paddy

☐ (2) Wheat

☐ (3) Sugarcane

☐ (4) Pulses

☐ (5) Vegetables

☐ (6) Oilseeds

☐ (95) Other crop

G5(TGXT). Specify the name of the other crop

G6. Why did you not apply Zinc on your land?

(select all that apply)

☐ (1) Land is far away

☐ (2) Not needed on my land

☐ (3) Zinc had a negative impact on my yield

☐ (4) Application costs are huge

☐ (5) Don’t have time for farming

☐ (95) Other

G6(TGXT). Specify other reason.

H1. In which of the following ways do you think the micronutrient subsidy scheme can be made better?

☐ (1) Micronutrient should be supplied according to land owned by the farmer

☐ (2) The internet connectivity should be improved.

☐ (3) The tablets should be of good quality.

☐ (4) There should be more stock of the micronutrients to meet excess demand

☐ (5) The workload should not be just on 1 person

☐ (6) There should be a better system to identify eligible farmers

☐ (95) Others

H1(TEXT). Specify other.

H2. Please collect the GPS coordinates

Please wait for 15-20 seconds to capture location on the phone

1. Interviewers: Team ID

2. Interviewers: Write any notes that data analysis or researchers should know.
